# Optimizing risk‐reducing surgery and aspirin decision aids for Lynch syndrome carriers using the person‐based approach: A think‐aloud interview study

**DOI:** 10.1002/jgc4.70089

**Published:** 2025-08-07

**Authors:** Kelly Kohut, Kate Morton, Lesley Turner, Rebecca Foster, Elizabeth K. Bancroft, John Burn, Emma J. Crosbie, Mev Dominguez‐Valentin, Mary Jane Esplen, Helen Hanson, Karen Hurley, Pål Moller, Neil Ryan, Katie Snape, CanGene‐CanVar Patient Reference Panel, International Lynch Decision Aid Stakeholder Panel, Caroline Dale, Sue Duncombe, Rochelle Gold, Sonia Patton, Warren Rook, Richard Stephens, Lesley Turner, Frankie Vale, Helen White, Ivan Woodward, Steve Worrall, Julie Young, Munaza Ahmed, Lyndsy Ambler, Antonis Antoniou, Stephanie Archer, Ruth Armstrong, Elizabeth Bancroft, Kristine Barlow‐Stewart, Lily Barnett, Marion Bartlett, Julian Barwell, Dany Bell, Cheryl Berlin, Felicity Blair, Matilda Bradford, John Burn, Sarah Cable, Melissa Cambell‐Kelly, Dharmisha Chauhan, Ruth Cleaver, Beth Coad, Gaya Connolly, Gillian Crawford, Emma Crosbie, Victoria Cuthill, Tabib Dabir, Mev Dominguez‐Valentin, Eleanor Davies, Glyn Elwyn, Mary Jane Esplen, D Gareth Evans, Pia Fabricius, Andrea Forman, Kaisa Fritzell, Claire Giffney, Joana Gomes, Rebecca Hall, Helen Hanson, Menna Hawkins, Deborah Holliday, Roberta Horgan, Karen Hurley, Margaret James, Ros Jewell, Siobhan John, Victoria Kiesel, Anna Koziel, Anjana Kulkarni, Fiona Lalloo, Helen Liggett, Aela Limbu, Kate Lippiett, Anne Lowry, Manami Matsukawa, Ranjit Manchanda, Tracie Miles, Shakira Milton, Pål Møller, Kevin Monahan, Laura Monje‐Garcia, Gabriela Moslein, Alex Murray, Jennie Murray, Kai‐Ren Ong, Anbu Paramasivam, Alison Pope, Sarah Pugh, Imran Rafi, Gabriel Recchia, Nicola Reents, Neil Ryan, Sibel Saya, Raza Sayyed, Salma Shickh, Toni Seppala, Lucy Side, Katie Snape, Sian Smith, Tracy Smith, Dawn Stacey, Barbara Stayner, Eriko Takamine, Katrina Tatton‐Brown, Helle Vendel Petersen, Robert Volk, Karen Westaway, Nikki Warner, Jennifer Wiggins, Lisa Wilde, Jennet Williams, Catherine Willis, Elizabeth Winchester, Emma Woodward, Alice Youngs, Diana Eccles, Claire Foster

**Affiliations:** ^1^ Centre for Psychosocial Research in Cancer: CentRIC, School of Health Sciences University of Southampton Southampton UK; ^2^ South West Thames Centre for Genomics, Clinical Genetics Service St George's University Hospitals NHS Foundation Trust London UK; ^3^ Department of Clinical and Biomedical Sciences University of Exeter Medical School Exeter UK; ^4^ Patient and Public Collaborator Southampton UK; ^5^ Urology Genetics The Royal Marsden NHS Foundation Trust Sutton UK; ^6^ Oncogenetics Team The Institute of Cancer Research Sutton UK; ^7^ Faculty of Medical Sciences Newcastle University Newcastle upon Tyne UK; ^8^ Division of Cancer Sciences, St Mary's Hospital University of Manchester Manchester UK; ^9^ Tumour Biology Department, Institute for Cancer Research Oslo University Hospital Oslo Norway; ^10^ Department of Psychiatry, Faculty of Medicine University of Toronto Toronto Ontario Canada; ^11^ Peninsula Clinical Genetics Service Royal Devon University Healthcare NHS Foundation Trust Exeter UK; ^12^ Stanford R Weiss, MD Center for Hereditary Colorectal Neoplasia Cleveland Clinic Cleveland Ohio USA; ^13^ Department NEON‐S, Faculty of Medicine and Health Technology Tampere University and TAYS Cancer Centre Tampere Finland; ^14^ Department of Gynaecological Oncology Royal Infirmary of Edinburgh Edinburgh UK; ^15^ Centre for Reproductive Health, Institute of Regeneration and Repair University of Edinburgh Edinburgh UK; ^16^ Faculty of Medicine University of Southampton Southampton UK

**Keywords:** cancer, decision making, genetics, oncology, patients, psycho‐oncology

## Abstract

Lynch syndrome “carriers” carry a germline pathogenic variant conferring gene‐, sex‐, and organ‐specific increased cancer risks. They are presented with difficult, interrelated choices over their lifetime. This study was part of a larger project to codesign a health intervention, Lynch Choices™ https://canchoose.org.uk to provide an information hub and decision support for carriers, their family members, and clinicians. This study aimed to answer the research question: What content, framing, and design elements of a decision aid for genetic cancer risk management are important to Lynch syndrome carriers? Adult carriers were invited to a think‐aloud interview to hear their thoughts about a prototype version of Lynch Choices™ containing values‐clarification exercises. The first half of interviews focused on the gynecological risk‐reducing surgery and the second half on the aspirin decision aid. Twenty carriers (eight men) were interviewed, half of whom had a personal history of cancer. Iterative refinement of Lynch Choices™ content and design was completed between interviews using a transparent table of changes from the person‐based approach. Following the interviews, reflexive thematic analysis was applied to the entire qualitative dataset. Three themes were constructed to guide further optimization and make recommendations for improved cancer risk communication in clinical practice. The three themes were: (1) Interpreting gene‐specific cancer risks and “What does it mean to me?”; (2) Words matter: Careful phrasing is important to feel understood; (3) Decision aids: They can help but might trigger emotions. Think‐aloud interviews provided in‐depth insight into the psychosocial context of carriers. This informed optimization of the decision aid to support engagement and promote shared decision making with healthcare professionals. The learning from this study had broader implications beyond decision aid development, to understanding preferences, needs, and experiences regarding genetic cancer risk communication and decision support.

**What is known about this topic**

Management choices regarding increased genetic cancer risks are complex and personal. Gaps in information and decision support for Lynch syndrome carriers have been found. Well‐designed decision aids can provide clearer, freely available, meaningful information. Routine use could promote better communication and engagement in shared decision making. However, a lack of decision aids has been found for genetic cancer risk management of carriers post‐genetic testing.

**What this paper adds to the topic**

This think‐aloud interview study aimed to address the research question: What content, framing, and design elements of a decision aid for genetic cancer risk management are important to Lynch syndrome carriers? Possible barriers to engagement were uncovered, which informed optimization. Clearer, easier‐to‐understand personalized cancer risk information was added. Values‐clarification exercises were carefully reworded to attend to the emotive nature of decision making. Clear links to clinical services were added to the decision aid, with the suggestion that a live hotline should be developed.

## 1 INTRODUCTION

Decision aids are tools to promote active involvement in healthcare decisions. Decision aids encourage people to think about what matters most to them, so they can make the right choice for their unique situation (see https://decisionaid.ohri.ca). Clarifying goals, values, and preferences is especially important for difficult decisions without a clear “right” answer or “right time” (Elwyn, 2021; Elwyn et al., 2009). Compared with usual care, decision aids can improve knowledge and accurate risk perception across a range of medical settings (Joseph‐Williams et al., 2021; Stacey et al., 2024), including genetic counseling about germline genetic cancer susceptibility (Kautz‐Freimuth et al., 2023).

Shared decision making, including the offer of decision aids, is recommended by national guidance (National Institute for Health and Care Excellence, 2021; NHS England, 2019). As patient‐facing resources that can be used at home or in clinics, decision aids present an opportunity to scale up delivery of information and decision support. This is especially needed in the context of genomic medicine, which is becoming embedded across medical services (NHS England, 2021). To identify and evaluate existing resources, a systematic literature review was completed (Kohut, Morton, Turner, et al., 2023). This revealed a lack of decision aids about genetic cancer susceptibility that could be readily implemented into current practice. Most were designed to support the decision whether to have genetic testing, with fewer resources regarding the downstream risk management decisions for carriers of a germline cancer predisposition(Kohut, Morton, Turner, et al., 2023). Genetic testing has moved into “mainstream” oncology, with routine screening of somatic tissue (tumors) to inform targeted treatment options (National Institute for Health and Care Excellence, 2017, 2020; Snowsill et al., 2014). There is not enough capacity in clinical genetics services to counsel all patients with cancer eligible for genetic testing. This has led to non‐genetics professionals offering testing at point of care in oncology clinics (Rumford et al., 2020). The number of patients identified as carriers of a germline cancer predisposition has increased at pace. Carriers and their families require support with choices that are personal, values‐laden, and time‐sensitive. Lynch syndrome is one of the most common germline cancer predispositions, estimated to be prevalent in about one out of 279 people (Win et al., 2017).

There are four Lynch syndromes that arise due to a constitutional (germline) **path**ogenic (or likely **path**ogenic) variant in one of the DNA mismatch repair (**MMR**) genes: *MSH2*, *MLH1*, *MSH6*, and *PMS2* or by deletion of the 3′ end of the *EPCAM* gene, which results in hypermethylation of the *MSH2* promoter (Ligtenberg et al., 2009). Collectively, these are referred to as ***path_MMR*** variants.^1^ Lynch syndrome “carriers” carry a *path_MMR* variant but may never develop cancer, especially with *path_PMS2* or *path_MSH6* variants, which confer lower risks (Holter et al., 2022). Testing all people with newly diagnosed colorectal or endometrial cancers, regardless of age or family history, identifies germline *path_MMR* variants in 3% and 6%, respectively (Peltomäki et al., 2023). Lynch syndrome is a lifelong, multi‐organ genetic cancer predisposition. Decisions may be “distributed” across time as they become relevant, involving consultations with clinicians across integrated care pathways and with significant others who influence decision making (Rapley, 2008). Decision aids could be particularly useful for carriers as they consider complex, interrelated decisions. These may be revisited as risks become more relevant with age or personal priorities change. Clearly designed decision aids could double as an educational resource for clinicians, friends, and family, alleviating some of the personal burden on carriers to inform others about their condition (Lloyd et al., 2022; Warner et al., 2022; Zhao et al., 2024).

To meet a clinical need to support Lynch syndrome carriers with decision making, the research team (KK, KM, RF, LT, DE, CF) codeveloped a digital decision aid called Lynch Choices™ (https://canchoose.org.uk). This took place as part of the Cancer Research UK‐funded program, CanGene‐CanVar (https://www.cangene‐canvaruk.org). A codesign approach (Grindell et al., 2022; Kohut et al., 2023) was used, partnering with a Patient Reference Panel (12 members with varied experiences with cancer and/or genetic testing) and a panel of international partners. The person‐based approach was used as a flexible set of methods combining in‐depth research with patient and public involvement and engagement (Yardley et al., 2015). Codevelopment was guided by the International Patient Decision Aids Standards (IPDAS) (Joseph‐Williams et al., 2014; Witteman, Maki, et al., 2021), Coulter's framework for decision aid development (Coulter et al., 2013), and the Medical Research Council guidance for developing and evaluating complex interventions (Skivington et al., 2021, 2024). Lynch syndrome was chosen due to the lack of available decision aids for carriers (Kohut, Morton, Turner, et al., 2023). Other factors included a timely National Lynch Syndrome Transformation Project in England aimed at improving education and testing pathways (Monahan et al., 2023), with similar initiatives internationally (Barrus et al., 2022; Kang et al., 2020) leading to a growing need for public‐facing support resources. Learning from the process of codesigning Lynch Choices™, including identification of core components, program theory, and guiding principles, will inform adaptation of the template to create other decision aids (Morton et al., 2022).

**FIGURE 1:**
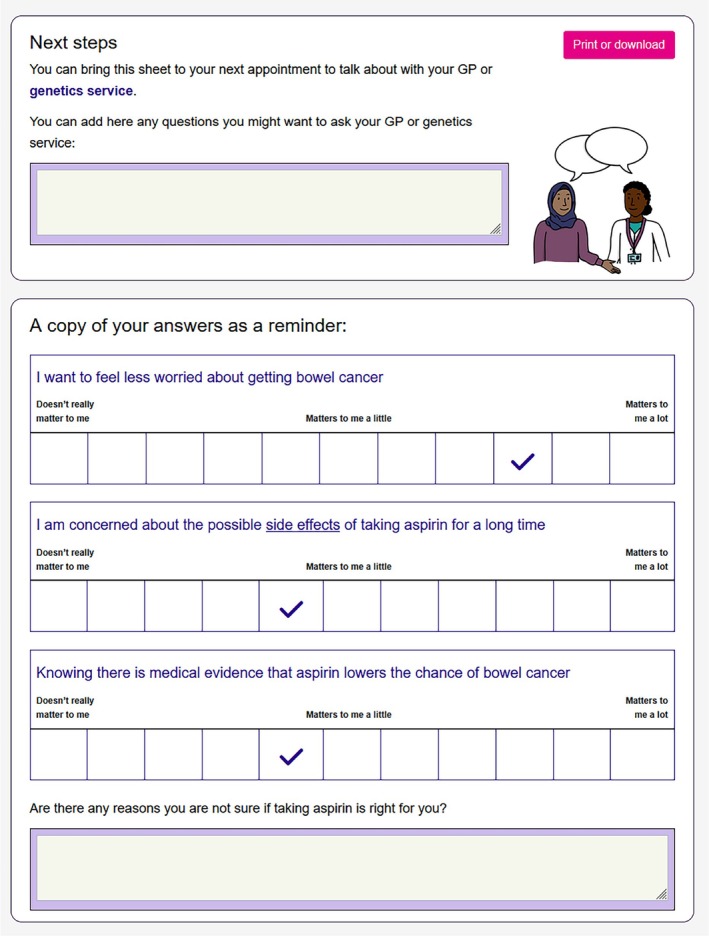
Excerpt showing results from optional values‐clarification exercise available for Lynch syndrome carriers to consider aspects of decision making and how much they matter to them at the time. A printable summary can be produced to bring along to discuss with a healthcare professional. This includes a free text box to note questions and a summary of the answers about how much aspects of the decision matter to someone, for example, “I want to feel less worried about getting bowel cancer.”

Codesign of Lynch Choices™ has been described elsewhere (Kohut et al., 2023, 2024; Kohut, Morton, Turner, et al., 2023). In brief, Lynch Choices™ is an interactive information hub and decision aid informed by theory and empirical evidence that is compatible with smartphones, computer screens, and tablets. Personalized, age‐stratified cancer risk estimates are accessible in graphical form via a unique link with the Prospective Lynch Syndromes Database (Dominguez‐Valentin et al., 2023) (PLSD, https://plsd.eu). Visual presentations of risk such as icon arrays are used. This is in line with best practice recommendations for communicating health risks using visual aids (Lipkus, 2007; Trevena et al., 2013). Two decision aids are included regarding cancer risk‐reducing options: “taking aspirin” and “operation to remove womb (uterus) ± ovaries.” Side‐by‐side grids compare choices. An interactive value‐clarification exercise using Likert‐scale ratings asks people how much different factors matter to them when making the decision (Figure 1). A printable summary is designed to prompt personalized genetic counseling discussions. Additional sessions are available regarding “cancer screening,” “living with genetic risk of cancer,” “talking to family,” “lifestyle,” and “more support,” providing links to charity websites, peer groups, and patient stories/videos. In line with fuzzy trace theory (Biesecker et al., 2017; Reyna, 2008), minimal text is used, presenting the “gist” of messages on each page. People can choose to delve into more detailed information according to their preferences and interest.

It is important to understand the psychosocial context of people who will use decision aids to support engagement and consider how to optimize the complex intervention (Band et al., 2017). Well‐designed decision aids provide clearer, more meaningful information that can result in better decision support to promote improved health outcomes (Stacey et al., 2017). This think‐aloud interview study aimed to address the research question:

What content, framing, and design elements of a decision aid for genetic cancer risk management are important to Lynch syndrome carriers?

There were two study aims:

1. Inform optimization of the Lynch Choices™ decision aid to support engagement and promote shared decision making about genetic cancer risk management.
2. Inform broader implications regarding the preferences, needs, and experiences of carriers, relevant to genetic cancer risk communication and decision support in clinical practice.

## 2 METHODOLOGY

### 2.1 Patient and public involvement

The Patient Reference Panel for the CanGene‐CanVar program in which this study was situated helped optimize documents before the ethics application. For example, they reviewed wording of the flyer advertisement and participant information sheet. This has been described elsewhere (Kohut et al., 2024) and is summarized in Table S1 in Appendix S1.

### 2.2 Study design

A qualitative think‐aloud interview study design was used (Bradbury et al., 2018). This provided in‐depth engagement with carriers looking at the prototype Lynch Choices™ in real time (Gorman et al., 2022; Grimmett et al., 2019; Howell et al., 2023; Morton et al., 2021; Pinto et al., 2023; Wolcott & Lobczowski, 2021; Yardley et al., 2010), in a protected time and safe space, from their socially constructed perspective (Ritchie et al., 2014). Interviews were conducted in batches in line with the person‐based approach, a set of methods to guide meaningful behavioral health intervention development (Bradbury et al., 2018; Morton et al., 2021; Yardley et al., 2015). This allowed iterative refinement of the prototype decision aid before moving on to the next batch of interviews (Sandelowski, 1995). Active listening to verbalization of cognitive processes provided the researcher with a situational observation of how carriers interacted with the decision aid. These qualitative data complemented other feedback gathered from partners (Kohut et al., 2024) to inform optimization of Lynch Choices™. Reflexive thematic analysis applied to the entire qualitative dataset was used to construct an understanding of the broader implications for clinical practice, beyond refinement of the decision aid.

Ethical approval was received from the study sponsor, University of Southampton, the National Research Ethics Service, and the Health Research Authority (REC reference 22/NI/010, IRAS Project 312473). The study was adopted onto the National Institute for Health and Care Research Clinical Research Network Portfolio. The Reflexive Thematic Analysis Reporting Guidelines (RTARG) were consulted to improve reflexive reporting (Braun & Clarke, 2024a).

### 2.3 Recruitment

People were eligible to take part if they were aged 18 or older and self‐reported as Lynch syndrome carriers. They were offered remuneration for their time in the form of a £30 voucher redeemable at https://www.amazon.co.uk. Two approaches were used for recruitment:

1. The PhD student/lead researcher (KK) is also the Lead Genetic Counselor at the South West Thames Centre for Genomics, which covers a catchment of 4 million people and is based at St George's University Hospitals NHS Foundation Trust in London, UK. KK invited the team of 15 Genetic Counselors and Consultants to inform eligible patients seen in clinics during the recruitment period about the study. A Research Practitioner screened the service register (database) to confirm *path_MMR* variant carrier status for the recruitment screening log. Invitations were sent in small batches to manage responses and avoid disappointment. Along with a verbal explanation by the clinician, a standard invitation letter was used (Appendix S1).
2. A convenience selection of people from outside the South West Thames service were invited to increase diversity in terms of participant characteristics and the geographical areas where clinical services were accessed. They had taken part in unrelated public engagement workshops organized via the Lynch Syndrome UK patient charity (https://lynch‐syndrome‐uk.org) by a member of the partner panel (JB) and agreed for their contact details to be used for future research. A flyer was sent to all 10 people on the list by the researcher. They were not screened for eligibility but were asked during interviews about their experience with genetic testing to confirm they were Lynch syndrome carriers. A flyer was sent via email by KK (Appendix S1).

People who expressed interest by email or phone were sent the Participant Information Sheet and Consent Form (Appendix S1) via email or post by KK or the Research Practitioner. People were given at least 24 h to review these documents before being contacted by KK to confirm if they wanted to take part and, if so, schedule the interview at their convenience.

A recruitment target of 20 participants was identified in advance as a pragmatic sample size in line with timescales and available time and resources (Malterud et al., 2016). Many other factors can be relevant to the selection of a target sample size for qualitative interviews, such as the availability of participants, complexity of the research question, quality of the data generated, and the nature of analysis (Malterud et al., 2016; Sandelowski, 1995). The target of 20 participants was confirmed as appropriate through iterative review of the interview transcripts, with confirmation that no new major concerns were raised about the prototype decision aid (Braun & Clarke, 2024a). Recruitment was completed following review of the final batch of interviews, which suggested that the decision aid modifications implemented so far had successfully addressed key issues with engagement, in line with the person‐based approach (Band et al., 2017; Bradbury et al., 2018; Yardley et al., 2010).

### 2.4 Data generation

Interviews were offered online, by telephone, or in person. At the beginning of the interviews, KK confirmed verbal consent and asked if participants had any remaining questions about the consent form. The positionality of the PhD student researcher (KK) was acknowledged to increase transparency about the power dynamic, including self‐awareness and reflexivity about challenging this (Braun & Clarke, 2024a; Farr et al., 2021). As a Genetic Counselor, KK brought many transferable skills to conduct data generation in the interview setting. These included establishing rapport, attention to emotions, respect for autonomy, active listening, and tailored interpersonal communication about complex genetic information (Biesecker, 2020; Sexton & James, 2022; Veach et al., 2007; Wainstein et al., 2023). KK's background influenced data generation through choice of question wording, use of silence or positive reinforcement, and prompting for fuller responses about selected topics regarding the research question. KK co‐constructed new knowledge together with participants through an active partnership with a constructivist approach (Braun & Clarke, 2024a; Crotty, 1998a).

Constructivism proposes that knowledge is gained by individuals through interactions with their social world, creating meaning for themselves as they move through life (Crotty, 1998b). The constructivist worldview applied to this study incorporated the ontological view that there are multiple realities and the epistemological standpoint that the researcher plays an active role in learning about these realities as experienced in personal context.

Each interview began with semi‐structured questions to help understand the context of participants' experiences as carriers and as a warm‐up for them to begin talking. These introductory interviews were reported elsewhere (Kohut et al., 2024). The second component of the interviews was the think‐aloud protocol during which participants looked at the decision aid, reported here (guide in Appendix S1). Participants had the option to do the interview in two parts.

The first half of think‐aloud interviews took place August 2022 to January 2023 and focused on reviewing the hysterectomy decision aid. The second half took place January to February 2023 and focused on the aspirin decision aid, which had been refined by applying any relevant changes from optimization of the hysterectomy session. Participants were invited to share their screen and navigate through the decision aid or ask KK to do this. Mostly open questions were used such as “Can you tell me what you think about this page?” to allow time and space to share “in‐the‐moment (i.e., online) thought processes” (Wolcott & Lobczowski, 2021). Simple probing questions such as “Can you say a bit more about that?” and “What made you feel that?” were used to clarify or elicit deeper understanding of meaning and contextual factors (Bradbury et al., 2018; Ritchie et al., 2014, 197–198; Yardley et al., 2015).

Interviews were audio‐recorded. Transcripts were created using automated software followed by manual error correction and removal of personally identifiable information such as names of participants or hospitals. Transcripts were pseudo‐anonymized with an identifier assigned: p001–p020. Demographics and questions to assess health literacy levels (Chew et al., 2004) were collected at the end of the interview to allow time for rapport‐building first.

### 2.5 Data analysis

The person‐based approach to intervention development (Bradbury et al., 2018; Morton et al., 2021; Yardley et al., 2015) enabled an agile response to participant feedback to optimize Lynch Choices™. Quotes from the think‐aloud interview transcripts were categorized into positive, neutral, or negative comments and entered line‐by‐line into a Table of Changes using Microsoft Excel by KK. Comments were assigned a prioritization category using **M**o**S**Co**W**: **M**ust have, **S**hould have, **C**ould have, or **W**ould like if resources allow (Bradbury et al., 2014). The researchers reviewed whether possible refinements to content and design were perceived as necessary and in line with the guiding principles developed using the person‐based approach (Morton et al., 2022), the IPDAS guidelines (Hoffmann et al., 2021; Joseph‐Williams et al., 2014), evidence from clinical guidelines, and the literature. Review took place with an experienced researcher (KM) initially after two interviews to reflect on findings and prioritize possible changes to the prototype. Reviews were then extended to take place after each batch of five interviews. Straightforward modifications were sent directly to the web developer to implement, whilst more complex issues were flagged for discussion with the wider research team, partners, and/or the CanGene‐CanVar Patient Reference Panel. Experts in the research team (DE, CF) and partners with specialist expertise were consulted, for example, regarding gene‐specific cancer risks (PM, MDG), clinical management guidelines (HH, KS), gynecological surgery (NR, EC), aspirin (JB), and psychosocial care (EB, MJE, KH).

The complete think‐aloud interview dataset was subsequently analyzed using reflexive thematic analysis (Braun & Clarke, 2006, 2019, 2022, 2024a) as an interpretive method (Braun & Clarke, 2024a). Themes were constructed regarding cancer risk management communication, framing, and design elements important for the wider community of Lynch syndrome carriers to support decision making. One PhD student researcher (KK) undertook familiarization with the transcripts through reading and rereading. Next, KK flexibly and creatively produced codes (Braun & Clarke, 2021) using the comment function in Microsoft Word. A mainly inductive approach was used, with some deductive analysis to ensure meaning co‐created with participants was relevant to the research question (Byrne, 2022). KK actively interpreted the codes, then generated and refined themes. These were reviewed with the research team (KM, RF, DE, CF) with discussion leading to further refinement by KK (Braun & Clarke, 2024a; Byrne, 2022). Qualitative research methods were prioritized to construct an understanding of varied perspectives from in‐depth interviews (Creswell, 2018), consistent with the constructivist stance that individuals have subjective experiences of their realities. As applied to research methods, constructivism acknowledges that researchers inevitably play an active role in constructing meaning when analyzing qualitative data (Ritchie et al., 2014). KK's social context, personal views, and experiences influenced how data were generated in the interviews, for example, how questions were worded and what prompts were used to elicit fuller responses (Malterud et al., 2016). This could also have influenced how much and what information participants felt comfortable to share (Braun & Clarke, 2023a; Byrne, 2022). KK was self‐reflective about how background and worldview influenced the way that themes were contextualized and interpreted during data analysis (Braun & Clarke, 2023b; Byrne, 2022). This was regularly reflected upon in meetings with the research team and transparently disclosed when reporting on participants' experiences (Braun & Clarke, 2022, 2024a).

Positionality and perspectives of the KK are acknowledged (Braun & Clarke, 2023b), as someone who identified as a cisgender woman of White race in a position of power as a healthcare professional involved in developing decision aids and other patient resources. However, the interviews were completed from the standpoint of a PhD researcher who was not responsible for the clinical care of the participants. This was explained in the study materials and introductions to the interviews.

## 3 ANALYSIS

### 3.1 Participant characteristics

Participant characteristics have been described elsewhere (Kohut et al., 2024). Aggregated characteristics are presented in Table 1. In brief, 20 carriers were interviewed for 46 min on average (range: 26–61 min), mostly by video call with one in person. Half had a personal history of cancer (*n* = 10). Twelve identified as women and eight as men. Two men reviewed the hysterectomy decision aid. They did not have a uterus or ovaries; therefore, they would not need to decide about hysterectomy themselves. However, they did mention relatives considering hysterectomy or who needed to decide about this in the future. Therefore, the men were able to make comments with this in mind.

**TABLE 1:** Characteristics of total sample of interview participants, consisting of *n* = 20 Lynch syndrome carriers.

	Number	% of total participants
1. Where did you hear about the Lynch choices website and booklet?		
a. Genetics service	10	45%
b. Cancer/oncology service	3	25%
c. General practitioner (Family doctor)	0	0%
d. Lynch syndrome UK	4	40%
e. Bowel cancer UK	0	0%
f. A friend or family member	0	0%
g. Other (please state here):	5	23%
2. How old are you?		
18–25	1	5%
26–40	6	30%
41–60	11	55%
61–70	2	10%
Over 70	0	0%
3. When did you leave full time education? (if you are still in education, please select the stage you are at now)		
Before finishing school	1	5%
After finishing school	6	30%
After finishing university	8	40%
After postgraduate studies	5	25%
4. What is your gender identity?		
Man	8	40%
Woman	12	60%
Other/Prefer to self‐describe: (optional to add text):	0	0%
Prefer not to say	0	0%
5. What is your ethnic group?		
Black Caribbean	0	0%
Black African	0	0%
Black Other	0	0%
Bangladeshi	0	0%
Chinese/South East	0	0%
Indian	2	10%
Pakistani	0	0%
White British	14	70%
Any other White	2	10%
Asian	2	10%
Other	0	0%
Prefer not to say	0	0%
6. Have you ever been diagnosed with one or more cancer?		
No	10	48%
Bowel (colon) cancer	8	38%
Endometrial (womb/uterus) cancer	0	0%
Other (please state here):	3	14%
7. What is your postcode (or country if outside the UK)?		
UK postcode	19	95%
Not answered	1	5%
Other country	0	0%
8. Do you have any access requirements you would like me to be aware of?		
Yes	2	10%
Not answered	1	5%
No	17	85%
9. How often does someone (like a family member, friend, hospital/clinic worker, or caregiver) help you read hospital materials?		
(1) Always	0	0%
(2) Often	0	0%
(3) Sometimes	0	0%
(4) Occasionally	2	10%
(5) Never	18	90%
10. How confident are you filling out medical forms by yourself?		
(1) Extremely	13	65%
(2) Quite a bit	6	30%
(3) Somewhat	1	5%
(4) A little bit	0	0%
(5) Not at all	0	0%
11. How often do you have problems because you find it difficult to understand written information about medical conditions?		
(1) Always	0	0%
(2) Often	0	0%
(3) Sometimes	3	15%
(4) Occasionally	8	40%
(5) Never	9	45%

Ten participants were recruited from the South West Thames clinical genetics service, and 10 were recruited from previous unrelated engagement workshops via Lynch Syndrome UK. Sixteen additional people were approached but did not respond, and two more consented but did not schedule an interview, giving an overall response rate of 20/38 (52.6%). All participants chose to proceed to the second, think‐aloud portion immediately after the introductory semi‐structured interview.

### 3.2 Intervention optimizations

#### 3.2.1 MoSCoW prioritization to inform iterative refinement

Examples of think‐aloud interview quotes classified into positive, negative, and neutral comments with **M**o**SC**o**W** prioritization regarding possible refinements to the prototype of Lynch Choices™ are shown in Table 2, with additional quotes in Table S2 in Appendix S1. Refinements that were implemented based on interview data included some that were non‐controversial and considered “**M**ust have” even if only suggested once, such as clarification of risk (see Appendix S1 for examples). This included clarifying information about ovarian‐ and endometrial‐specific cancer risks. Clearer pictures of the organs were added. Another change involved putting lifetime risks for colorectal cancer in a separate section from the 10‐year risks, to avoid confusion when trying to compare these. The researchers paused after 10 interviews to optimize the aspirin decision aid prototype as much as possible. This way, relevant changes could also be applied to the hysterectomy decision aid before asking participants to engage with it. The number of refinements reduced after each batch of interviews. This suggested subsequent participants found the refined version of Lynch Choices™ easier to understand and navigate. The researchers were satisfied that qualitative data had provided sufficiently rich description (Bradbury et al., 2018; Braun & Clarke, 2024a; Sandelowski, 1995) to provide information power (Malterud et al., 2016).

**TABLE 2:** Excerpt from table of changes showing examples of how comments from think‐aloud interviews were categorized into negative, neutral, and positive.

ID	Negative comments	Neutral comments	Positive comments	Possible change	MoSCoW
P003	It might be worth saying with [HRT] tablet about disadvantages. If you've got compromised bowel absorption then you're not gonna absorb it. I can't think of a time where it would be more appropriate to take a tablet… I guess it's probably cheaper. You're supposed to rub [gel] on the top of your arm or inner thigh		That's really good. Vaginal estrogen as well. That's a good picture of that I think	Explain tablets less often prescribed because of the chance of blood clots. Note people who have had bowel surgery may not be able to take tablets. Change picture of gel showing where it is applied. Checked with expert stakeholders	Must have
P004	Most people get these symptoms daily, like IBS, so many people are like, I'm so bloated today. I ate bread. A lot might read this and go, oh my God, I've got cancer. That might not be the case. I mean, I would read that and be like, wow, I have all of those things			Add: “Many people may find these symptoms are normal for them due to other reasons. The important thing is to watch out for any changes that are not normal for you, or do not go away.”	Must have

*Note*: Possible decision aid modifications were identified and prioritized using MoSCoW. This addressed the research question regarding what content, framing, and design elements of a decision aid are important to Lynch syndrome carriers. Optimization of the decision aid was completed to support engagement and promote shared decision making about genetic cancer risk management (study aim 1).

#### 3.2.2 Reflexive thematic analysis to inform optimization and implementation

Three overarching themes were constructed from the entire qualitative dataset after completion of the interviews (Table 3). KK's prior knowledge and experience as a clinical genetic counselor, healthcare leader, and decision aid developer actively influenced data analysis during construction of themes and recommendations (Braun & Clarke, 2022, 2023b; Byrne, 2022). A focus on shared meaning made reflexive thematic analysis an appropriate methodological approach (Braun & Clarke, 2023a). KK attempted to remain empathically neutral throughout data collection, interrogation, and reporting, seeking to understand and care about participants' views whilst remaining impartial and reserving judgment as much as possible (Ritchie et al., 2014). The positionality of the research team will have also influenced analysis and reporting and is acknowledged: All are cisgender women of White race. KM and RF are post‐doctoral health researchers experienced in developing health interventions. LT is a patient advocate with a prior history of breast cancer, involved in decision aid development. DE is a consultant geneticist and leading researcher in risk communication and germline genetic cancer predisposition. CF is a health psychologist and leading researcher in the psychosocial aspects of the impact of cancer and managing this impact with behavioral health interventions.

**TABLE 3:** Results of reflexive thematic analysis on the entire qualitative dataset from think‐aloud interview transcripts.

OVERARCHING THEME: Narrative summary	Subtheme	Exemplar quotes from *n* = 20 interview transcripts
1. INTERPRETING GENE‐SPECIFIC CANCER RISKS AND “WHAT DOES IT MEAN TO ME?”: Lynch syndrome carriers were often unaware about current gene‐specific clinical guidelines for cancer risk management. Some did not know which gene was relevant for their family. Several found the personalized, age‐stratified visual presentations of cancer risks in Lynch choices helpful. These seemed to improve understanding and place risk into context considering personal factors such as age	1.1 Gene‐specific cancer risks are not well understood	“Do all of those genes all affect the same parts? Sorry, just before I carry on, I just had a thought coming to my mind. So can you carry 2 of those gene or 3 of them or all 4?” (p010)
	1.2 Age‐stratified cancer risks help to put decision making into context with other competing priorities	“It's interesting that it says you are higher risk if you are older. How old does that go to? Because I don't have some tests anymore because I'm past that age.” (p009)
	1.3 Visual presentations including pictures and icon arrays were useful and people had preferences, for example, about shape, color, and lifetime vs. 10‐year risks	“That's the sort of thing that makes it so much clearer. You hear all these percentages, but actually visually you see it like that. I'm a really visual person. You can see the impact it's having if you take the aspirin. Look at that.” (p018)
2. WORDS MATTER: CAREFUL PHRASING IS IMPORTANT TO FEEL UNDERSTOOD: Lynch syndrome carriers had emotive reactions to the words presented in Lynch choices about risk‐reducing options. This prompted consideration of personal values and priorities. This process could encourage deliberative decision making. It was important for some carriers to feel their rationale for deciding or waiting to decide was reflected in the language used in the decision aid	2.1 Choice of words to describe decisions needs to be sensitive and done in partnership with the people who will use the resource	“Probably one of the biggest reasons apart from having a family, why I am not choosing to have this yet is cause I am not looking forward to menopause, horrible side effects. So that would be a huge decision‐maker for me. I would almost be like, should I just live with my risk of cancer as opposed to bringing this about early? Like that's a big thing, I think.” (p004)
	2.2 Symbols and icons are preference‐sensitive and benefit from codesign	“I think that's really powerful. It's kind of scary… I knew the risks were higher than the general population, but it's putting it into this graphic with people [icon array] that hammers it home.” (p004)
3. DIGITAL DECISION SUPPORT INTERVENTIONS: THEY CAN HELP BUT MIGHT TRIGGER EMOTIONS: Lynch syndrome carriers experienced gaps in information, understanding, and support. Lynch Choices showed potential to help to fill some of these gaps. However, there were some specific clinical questions raised by looking at the decision aid. These were directed back to clinical services. Links to clinical genetics, specialist care, other resources, and charities needed improvement	3.1 Engaging with the intervention can enhance understanding but may make carriers feel anxious or confused about their cancer risks and options	“Makes you stop and actually question those points. Your mind goes into overdrive. And there's so much to consider, especially when you have received that diagnosis.” (p018)
	3.2 Carriers would benefit from better links to specialist information, care, and referrals	“It's impossible to get an appointment. I've gone to private healthcare because I cannot speak to a GP [General Practitioner]. If there are others like me that have moved since their diagnosis, they might be in the same place.” (p004)
	3.3 Carriers highlighted other information and resources like charities and peer support that they found helpful and thought should be signposted	“These are statistics and there are charities that can help. Just a couple of hyperlinks I think would be really nice.” (p019)
	3.4 Links to clinical genetics services may be lost over time and need review	“I don't even know who my genetics service are. I haven't spoken to them in 15 years. Now I live in a complete different part of the country, I don't know who my local genetic service would be.” (p004)
	3.5 Lynch choices as a resource was helpful and valued by Lynch syndrome carriers	“I think it's really good, there's a lot of information now, which is great, 10 years ago there was nothing. But this is amazing. There's so much information, which probably took me a very long time to get. And this is great ‘cause it's all in one place’.” (p017)
	3.6 Digital decision support resources should ideally include a link to speak to someone for help, for example via a hotline	“What if you're quietly panicking inside and you've just been on the website? A contact you could ring? I don't know if there's people you could speak to. I'm fortunate that when I've needed somebody [genetic counselor] has been there <laugh>.” (p018)

*Note*: Three overarching themes are presented, with narrative summaries and subthemes. Exemplar quotes are included for each subtheme. Findings regarding the preferences, needs, and experiences of carriers had broader implications beyond optimization of Lynch choices. These findings led to recommendations to improve genetic cancer risk communication and decision support in clinical practice (study aim 2).

Table 3 summarizes the themes with analytic narratives, subthemes, and exemplar quotes, with additional quotes in Table S3 in Appendix S1. The themes are further developed below, exploring how the qualitative data addressed the research question: What content, framing, and design elements are important to Lynch syndrome carriers? These data informed optimization of the decision aid (study aim 1) and had broader implications to recommend improved genetic cancer risk communication and decision support in clinical practice (study aim 2).

#### 3.2.3 Theme 1. Interpreting gene‐specific cancer risks and “What does it mean to me?”

Carriers, especially those diagnosed many years ago, could be unaware of important research findings leading to more precise gene‐, age‐, sex‐, and organ‐specific risk penetrance estimates for Lynch syndrome‐related cancers. These findings have led to the development of distinct management recommendations for the four Lynch syndromes (Møller et al., 2023) (see https://www.ukcgg.org).

I didn't realise, depending on which type of Lynch [path_MMR variant in which gene], you could have just your womb [uterus] out or they take the ovaries [as well]. (p001)

If people were not sure in which MMR gene their familial pathogenic variant was found, this could lead to confusion and anxiety when asked to select the gene or “I don't know” from a drop‐down list to view their personalized cancer risks and corresponding healthcare choices.

I don't even know. I don't even have my gene there, so that would stump me. I feel like mine is none of those… if mine was in the list, I'd recognise it, but it's not any of those [Note: all the Lynch syndrome‐related MMR genes were shown]. (p004)

Guidance was added to contact their genetics service if carriers did not know which gene was relevant to them. A pop‐up message was created to appear when hovering over the words “genetics service” to suggest asking their General Practitioner (GP/family doctor) for a referral if needed.

Some carriers valued visual presentations to help understand their chances of developing Lynch syndrome‐related cancers, compared with the average population risks. The risk‐reducing effect of interventions such as aspirin or hysterectomy was displayed by showing the proportion of carriers expected to develop cancer if they completed these interventions versus if they did not.

I think it's very good to visualise it, because numbers don't… but when you see a visual, I think it helps. (p005)

For example, participants appreciated the use of icon arrays. One noted some preferences regarding shape (preferring outlines of non‐gender‐specific people instead of dots) and color:

The blue people [icon] is good. (p013)

Details about age‐related cancer risks could be important to help participants draw meaning for themselves. To do this required thinking about which risks were relevant to carriers at various life stages. Graphs displayed via the link to PLSD (https://plsd.eu) and color‐coded age‐stratified risks with icon arrays could help understanding and put risks into context.

I think this tells a… story saying your risks start to appear at 35, 40 [years old] and… [hysterectomy is] something you should consider. [Interviewer: So, you liked the one with the coloured people showing by which age?] I think I like this representation of it building out. I think that's a much more useful representation of risk [than simply displaying the absolute lifetime risk]. (p007)

Since both the participants and the researcher had high levels of health literacy, their numeracy skills were likely higher than the average population of carriers. It is not known how marginalized populations with less education would have engaged with and understood the Lynch syndrome‐related cancer risks. These are considered complex due to the multi‐organ, gene‐, sex‐, and time‐specific risks, preventing one simple message from being sufficient to inform all carriers.

#### 3.2.4 Theme 2. Words matter: Careful phrasing is important to feel understood

In the option grid section of the decision aid, possible choices are compared, for example, to take aspirin daily or not to take aspirin daily. The choice of words was scrutinized by participants. Interacting with the decision aid could evoke strong reactions and feelings, which were holistic, not only pertaining to the decision at hand:

Having the operation [hysterectomy] also makes you feel lots of other things, not necessarily related to cancer. Maybe it should be ‘how does this make me feel about my cancer risk?’ [instead of, ‘How does this make me feel?’]. (p003)

Wording was changed as suggested to be specific to feelings about cancer risk.

Carriers expressed wanting to feel recognized, understood, and validated for their investment in deliberative decision making, whether they had already made a choice about a risk‐reducing intervention or were postponing this. One had a personal reason why they were waiting and did not want this to be perceived as “doing nothing”:

Why is it saying, ‘Don't have the operation’? So that's little help. It's just the fact that it says ‘do not’. It's the only thing I don't like. Cause, it's like somebody is telling me ‘do not have the operation’. Rather than not have the operation because you were happy [due to a valid personal reason for delaying, such as wanting to have a child first]. [It's] the wording… But otherwise, fine. (p001)

This led to altered phrasing with a third option added called “wait to decide.” This aimed to avoid disengagement or loss of trust in the decision aid if a carriers' personal choice was not represented.

The values‐clarification exercise involved rating how much factors mattered to carriers when deciding, for example, “not having to worry about getting cancer.” Wording of this was optimized based on participants' reflections. One participant described not wanting to be labeled as a “worried” person regarding a single decision. Their life context was more complicated than this. Other interrelated priorities needed to be weighed up at the same time. Some participants had struck a balance between being worried enough about cancer to accept that being a carrier would change their life choices but being able to cope and not let this worry take over their sense of self. There was more to carriers than their carrier status. One was able to put this into context in a healthy way:

I dunno, the wording… like I worried… I don't worry about it. I'm just, I want to do something to reduce my chances of getting it [cancer], but on a day‐to‐day basis, I don't worry about it. (p011)

Decision aid refinements were implemented to suggest empowerment as a motivator over simply feeling less worried. Along with “I want to feel less worried,” the phrase was added, “I want to do something to help lower my chance.” This acknowledged the desire for people to act and feel more in control of their health.

KK's training in advanced counseling techniques and personal interest in learning how to best support and empower carriers may have encouraged the highly educated participants to share more. It is also possible that they shared less if they felt intimidated or had feelings that they thought did not agree with KK's. Carriers from marginalized communities were not included in the study. They may have reacted differently, which could have altered findings. This deserves further research.

#### 3.2.5 Theme 3. Decision aids: They can help but might trigger emotions

Carriers described navigating integrated healthcare pathways for surveillance and treatment over their lifetime. This spanned primary care (prescriptions for aspirin or hormone replacement therapy) and secondary care (colonoscopy, risk‐reducing surgery, or oncology treatment). Navigation required knowledge and tenacity, with specialist tertiary care services (genetics) a helpful and appreciated but overstretched coordinator of resources. For carriers who had moved or lost touch with their genetics service, learning about new recommendations or missing referrals that they did not know about prior to reading Lynch Choices™ could be alarming. One said:

I've never spoken to a gynaecologist in my life. I have no idea how to go about being referred apart from ringing my GP and, I'm not gonna lie, it's impossible to get an appointment. I've gone to private healthcare because I cannot speak to a GP. If there are other people like me that have moved since their diagnosis, they might be in the same place. I don't even know who my genetics service are. I haven't spoken to them in 15 years. (p004)

Emotive reactions suggested that a live chat function, hotline, or other quick and direct link to specialist services should ideally be added to Lynch Choices™:

What if you're quietly panicking inside and you've just been on the website? Actually having a contact that you could ring? I don't know if there's people you could speak to. You know, I'm fortunate that actually when I've needed somebody to talk to that [genetic counsellor] has been there <laugh>. (p018)

Live telephone or digital links to an on‐call specialist are beyond the scope of Lynch Choices™. This will be trialed in the future, should resources allow. A “More Support” section was added along with links on several pages about how to get in touch with genetics services or other clinical services. In the UK, a National Health Service (NHS) hotline (111) is available at all hours in case of distress or emergencies. This is noted on the NHS website, which is linked from Lynch Choices™ in several places. The study participants may have represented a self‐selected group that was quite proactive about obtaining health‐related information and had a stable life context that allowed them to volunteer for a long, in‐depth research interview. Other carriers with less agency or supportive life structure may require the live hotline link even more, since they could be more likely to become distressed by reading new or alarming information.

Participants gave their overall impressions of the website after reviewing the decision aid section in detail. This included other sections such as “more support” and “living with a genetic cancer predisposition.” Many positive and enthusiastic comments were expressed about Lynch Choices™ overall as a helpful, trusted, central source of information: “I love that it exists” (p001), “This will be a lifeline to so many people” (p002). However, one participant stressed it was important this did not replace lines of communication with healthcare professionals:

This shouldn't be seen as taking the place of speaking to a professional… I wonder if a disclaimer [is needed] that suggests this is to try and assist and guide and not a fail‐safe method of diagnosis. For some people this may be… extremely helpful to kickstart their understanding. (p012)

A disclaimer was added to the landing page. This noted that Lynch Choices™ was designed to be used with support from healthcare professionals. Decision aids may help people think about their choices at home before discussing them with a specialist.

## 4 DISCUSSION

This study enabled novel insight into the perceptions of Lynch syndrome carriers using a decision aid to consider choices regarding hysterectomy ± oophorectomy and taking daily aspirin. The think‐aloud interview technique enabled a real‐time, page‐by‐page glimpse into how participants engaged with the decision aid. This identified barriers to engagement and informed iterative refinement and optimization before implementation into clinical practice. Participants expressed enthusiasm about Lynch Choices™ and regret that something similar was not available for them when they needed it. Findings suggested potential for routine uptake of Lynch Choices™ to help prepare and encourage people to actively participate in values‐based, shared decision making about cancer risk‐reducing interventions. This is in line with the wider literature, which has demonstrated the benefits of many other IPDAS‐compliant decision aids across a range of medical settings compared to usual care (Stacey et al., 2024). Perhaps particularly for those diagnosed many years ago who had been given information that was out of date, the increased knowledge provided by Lynch Choices™ could be anxiety‐provoking. This suggested safety‐netting links to clinical services should be included, preferably with a live link to a professional, if resources allow. This could maximize potential to improve health outcomes by connecting carriers to a central, trusted source of online and in‐person support. Importantly, the website is interactive, with information tailoring. Support can be accessed in a personalized way at appropriate time points. More research is needed with marginalized populations including other ethnicities, lower literacy, visually/hearing impaired, and non‐English‐speaking. Projects with these communities will explore how they interact and react to the content, format, and decision of the decision aid to ensure it is relevant and meaningful to them.

The implications of each theme are discussed below, with reference to the wider literature.

### 4.1 Theme 1. Interpreting gene‐specific cancer risks and “What does it mean to me?”

Best practice guidelines for risk presentation (Bonner et al., 2021) were followed during early codesign of Lynch Choices™. These guided the inclusion of visual displays, absolute risks, common denominators, and comparison with baseline. In this case, increased genetic cancer risks were compared with average population risks. However, confusion and misunderstanding about gene‐specific cancer risks experienced by interview participants necessitated iterative refinement to content and design. It was not surprising that people struggled to understand their cancer risks and what it meant to them, given established challenges presenting risk information to the public (Freeman, 2019; Spiegelhalter, 2017). Evolving scientific knowledge from prospective studies has resulted in gene‐specific clinical management guidelines for the four Lynch syndromes. This makes it especially important that Lynch syndrome carriers understand their personal cancer risks. For example, risk‐reducing removal of ovaries is no longer recommended for *path_PMS2*, and colonoscopy surveillance begins later for *path_MSH6* and *path_PMS2* variant carriers (Dominguez‐Valentin et al., 2023; Møller et al., 2023) (see https://www.ukcgg.org).

Participants generally reported high levels of health literacy. Those with lower levels could experience even more difficulty with understanding (Gillian et al., 2015; Muscat et al., 2021). Since they were not included in the research, it is not known how this could have impacted their engagement with and understanding of the decision aid. This study began to address the gap in research regarding how carriers experience and interpret personalized genetic cancer risk estimates in a real‐world scenario (Trevena et al., 2021). The think‐aloud interview format provided a safe and protected space to gain insight about how to improve risk communication. Patient engagement is crucial to codevelop decision support resources that will have a positive impact on outcomes (Grossman Liu et al., 2021). Developers also need to monitor equitable allocation to avoid exacerbating health disparities for marginalized populations such as those with lower digital or health literacy (Killian et al., 2024).

Text and visual cancer risk presentation in Lynch Choices™ were refined based on participant feedback. In another think‐aloud interview study, people offered breast cancer screening expressed a desire for more personalized leaflets with risks described in percentages and links to information about modifiable factors (Gorman et al., 2022). They preferred positive reframing of risk (presenting the number of women *not expected* to develop breast cancer) for the icon arrays due to concerns about anxiety (Gorman et al., 2022). By contrast, participants looking at Lynch Choices™ liked the icon arrays presenting the number of people *expected to* develop cancer. This is in line with behavioral theories suggesting efficacy from demonstrating a serious threat to health (Atkins et al., 2017; Kahneman & Tversky, 1979; Michie et al., 2011) and other research into the effect of gain‐and‐loss framing on behavioral intentions (Keyworth et al., 2018; Okuhara et al., 2014). Personalized risk communication about Lynch syndrome‐related cancers is challenging due to the need to incorporate risks for multiple cancer types that are age‐ and sex‐specific and vary for the four Lynch syndromes (Dominguez‐Valentin et al., 2023; Møller et al., 2023). This was achieved in Lynch Choices™ by including a unique connection to the PLSD. This live database produces personalized cancer risk graphs. Cancer risks can be modified by other factors. Lynch Choices™ provides a comprehensive, “one‐stop shop” for information which includes a section on modifiable lifestyle factors, which warrant further research and integration into online risk calculation tools. Choices about genetic cancer risk management are intertwined. Carriers must decide upon these with a holistic view of their in‐the‐moment life context. Interpretation of gene‐specific cancer risks needs further exploration with other communities to avoid the website only being beneficial to more privileged carriers with higher numeracy skills who can make sense of graphs and percentages.

### 4.2 Theme 2. Words matter: careful phrasing is important to feel understood

Inclusion of explicit values‐clarification exercises in decision aids can reduce decisional conflict and increase values‐congruent choices compared with control conditions or implicit methods that do not involve interaction with anyone or anything (Witteman, Ndjaboue, et al., 2021). Lynch Choices™ includes interactive exercises asking carriers to explicitly clarify their values. They are asked to rate how many various factors matter to them related to the complex health decisions of having a hysterectomy or taking daily aspirin. Using multicriteria decision analysis (Thokala et al., 2016), a computer algorithm creates a summary page indicating how sure people appear to be about the decision. Listening to reactions in think‐aloud interviews about the choice of words presented in the values‐clarification exercises drew attention to the emotive nature of these decisions. This led to decision aid refinements to avoid disengagement (Binion et al., 2022).

Carriers choosing risk‐reducing interventions need to do so with the knowledge that they might never develop the relevant cancer(s). They must cope with the tension that comes from managing uncertainty (Han, 2021a, 2021b; Harrison et al., 2019) because it is impossible to know whether they will develop cancer in the future, and if so what type and at what age. There were strong emotions evoked by the idea of “doing nothing” presented in the decision aid. Some participants were committed to taking a decision but were not ready to follow through, for justifiable reasons. Readiness for decision making can be influenced by several elements including attitude and emotional distress, which are personal and can change over time (Keij et al., 2024). Simple refinement to include a third option: “wait to decide later if you're not ready now” was considered more tolerable to manage feelings of guilt, anxiety, and cancer worry. It was added to provide reassurance for participants who did understand their risk correctly and had an acceptable plan to deal with it at the right time. Another qualitative interview study reviewing a cardiac defibrillator decision aid also found that participants' understanding, and articulation of personal values was difficult and sensitive, leading to refined wording from asking about “values” to “what matters to you most?” (Carroll et al., 2018). Using careful phrasing, including simple language like this could help carriers to feel understood. Engaging with the decision aid to think about their values could help to promote personalized, shared decision making with their healthcare provider during clinical interactions (Elwyn et al., 2016).

### 4.3 Theme 3. Decision aids: they can help but might trigger emotions

Participants engaged in learning about their cancer risks and options when looking at Lynch Choices™. However, for some, this stirred up feelings of shock, cancer worry, and uncertainty about the best next steps. Findings were mirrored in a think‐aloud interview study of people offered breast cancer screening who reviewed risk communication materials, which also found that looking at these resources aroused strong emotions (Gorman et al., 2022). Lynch syndrome carriers have often already dealt with a personal and/or family history of cancer, which may have made them more accepting of language interpreted as “too scary” for people at average risk. The provision of good quality, easy‐to‐understand information could help manage emotions such as anxiety and uncertainty. This could help to shift people into a state of readiness for decision making, as found in a telephone interview study of Lynch syndrome carriers (Campbell‐Salome et al., 2021).

Robust evidence supports the potential to improve outcomes including knowledge, decision satisfaction, and preparedness through eliciting values‐congruent preferences with shared decision making complemented by decision aids (Birkeland et al., 2022; Montori et al., 2022; Rake et al., 2022; Steffensen et al., 2018; Witteman, Ndjaboue, et al., 2021). For carriers, decision making is not a one‐time event. They are asked to weigh up choices over time, as increasing cancer risks become relevant at certain ages. They may be dealing with other competing life events and priorities, such as family planning or a cancer diagnosis in themselves or relatives. This has parallels with broad frameworks about distributed (Rapley, 2008) and shared decision making using the Ottawa Decision Support Framework (Stacey et al., 2020). These are based on multiple theoretical constructs about how people make decisions over time, revisiting information or meeting with relevant people as they move through stages of decision making. A public‐facing, theory‐based complex intervention such as Lynch Choices™ could fill part of the information and decision support gap for carriers. Use of the decision aid could empower carriers to start their thought process around deliberative decision making. However, without consideration for context and easy access to support, there could be unintended consequences. These could include distress about cancer risks or disengagement from the decision aid (Skivington et al., 2021), leaving carriers to grapple with uncertainty on their own.

Carriers mostly expressed enthusiasm for using Lynch Choices™ at home. One made an important suggestion to add a “hotline” to speak to a real person for help and reassurance. Public‐facing risk communication materials coupled with a direct “hotline” to a clinical point of contact were also suggested in another study by interview participants offered breast cancer surveillance (Gorman et al., 2022). This approach has also been used to successfully streamline genetic testing for people with breast cancer (Torr et al., 2022). Other qualitative research involving people at increased genetic cancer risk also highlighted the need for accessible links to specialist genetics services to reduce the burden of navigating healthcare (Warner & Groarke, 2022). A decision aid, however, helpful, cannot, and should not replace clinical interactions, particularly as it has been found that people will often not contact clinical services when this is presented as optional (Høberg‐Vetti et al., 2016; Nilsson et al., 2019; Sie et al., 2014; Torr et al., 2022). The development of quality decision aids for carriers is needed to add to the web‐based platforms already in use to facilitate genetic testing decisions (Kohut, Morton, Turner, et al., 2023). Genetic cancer risk management decisions faced by carriers are higher in number and complexity compared with the one‐time decision of whether to have genetic testing. These choices involve consideration of emotions, behavioral preferences, and cognitive processes, with adaptation best supported by genetic counseling (Biesecker, 2020). The knowledge gained from this interview study supported offering decision aids for carriers of a genetic cancer predisposition to complement genetic counseling. Findings highlighted the importance of codesign with the people who will use the resource to support engagement and relevance. Alongside decision aids and other patient‐facing resources, carriers need access to specialist advice for coordination of care and timely referrals for surveillance or risk‐reducing interventions (Holter et al., 2022). Links should be built between the decision aid, clinical services, and other helpful community resources to make care more joined‐up, safer, and provide a better experience and decision satisfaction for carriers.

### 4.4 Limitations

All participants were Lynch syndrome carriers; however, although they looked at the decision aid and provided feedback in real time, this was not always a “real‐world” evaluation of their experience. They may have already made their choice and acted upon it (for example, already had hysterectomy), or it was not relevant to them (for example, not recommended to take aspirin due to being over age 70 years). Even for participants for whom the decision aid was currently relevant, the think‐aloud interview was by nature an artificial scenario. Some might not have been comfortable sharing all their thoughts in this setting. The positionality of the researcher as an experienced genetic counselor involved in developing the decision aid could have led to participants sharing more or less feedback and may have influenced their comments.

Participants had a range of characteristics, but not all groups were represented, including *path_PMS2* variant carriers with lower cancer risks, people with lower literacy levels, and those from marginalized ethnicities. To engage with these and other marginalized groups, additional funding will be sought for cultural translation of the decision aid, public engagement workshops, and research projects. This will involve trusted leaders to identify the preferred time, place, and method to invite people to share their lived experiences and preferences. For example, some might prefer a group setting in a relaxed location in the community (Hainsworth et al., 2022) or to be invited through community charities aligned with their identity (Shakirat Kekere‐Ekun et al., 2025).

Future studies will capture usage statistics and outcome data for the Lynch Choices™ decision aid, including knowledge, decisional conflict, decision satisfaction, anxiety, and distress. Impact evaluation will inform further optimization. This will also enable audit of characteristics to direct purposive recruitment of marginalized populations to research to investigate barriers to understanding or engagement. Consultation with screen‐reader users and experts in web content accessibility will be pursued to ensure the content is accessible by people who are blind or have other neuroprocessing differences requiring alt text descriptions accompanying visual images.

### 4.5 Reflections

Reflexive reporting encouraged consideration of how the research process was shaped by the active role and subjectivity of the researcher to co‐create knowledge with participants (Braun & Clarke, 2024a, 2024b). Quality, reliability, and validity were enhanced by regular reflections with the research team, the CanGene‐CanVar Patient Reference Panel, and expert partners to prompt the researcher to clarify ambiguities and create richer and more nuanced interpretations and reporting of the data (Mays et al., 2005; Morse et al., 2002).

The constructivist worldview allowed exploration of the proactiveness and self‐driven preferences (Neimeyer, 1993) for individual carriers acquiring knowledge and considering management choices about their genetic cancer risks. Limitations of constructivism include difficulty transferring learning from multiple realities, resulting in a lack of universality or application of one overarching theoretical framework (Dennick, 2016). As the researcher, KK acknowledged and reflected on positionality and privilege and how this could influence data generation and analysis. Qualitative methods with inductive analysis stayed close to the data, recognizing that experiences and preferences are likely to differ between participants and that decision making cannot be separated from social context (Popay & Williams, 1998). Interpretive reporting of themes explicitly acknowledged the personal nature of understanding cancer risks and decision making. Pragmatism was needed to synthesize knowledge about multiple realities from a constructivist viewpoint to make recommendations that could be applied in real‐world clinical practice on a large scale. Learning about different participants' realities encouraged a flexible approach to the codesign of the decision aid. Features like optional values‐clarification exercises encouraged more personalized decision making for a group of carriers with varied personal priorities and preferences.

### 4.6 Final considerations

The think‐aloud interviews provided novel insight into how a decision aid for Lynch syndrome carriers can be optimized to support engagement and promote shared decision making about genetic cancer risk management. Possible barriers to engagement were uncovered. This informed decision aid refinements to provide clearer, easier‐to‐understand cancer risk information. Values‐clarification exercises were carefully reworded to attend to the emotive nature of decision making. Links to clinical services were added throughout, with reminders that the decision aid was designed to complement interactions with healthcare professionals.

Participants included a varied group of carriers, including those tested many years ago who were unaware of recent developments or how to access genetics services and specialist referrals. People accessing Lynch Choices™ in the future could include those newly identified, their family members who have not yet been genetically tested, and others who locate the resource themselves online via charity or other websites searching for timely information. Therefore, it is important that the decision aid is easy to access and understand and includes signposting to sources of support.

Codevelopment of Lynch Choices™ included frameworks and toolkits to map project outputs, potential barriers, and alignment with healthcare priorities (Kohut et al., 2024). Guided by the person‐based approach (Yardley et al., 2015), real‐time studies with carriers and engagement with a group of expert partners were included. This maximized the likelihood that the decision aid will be accepted, easy to understand, useful, and engaging (Damschroder et al., 2009) before roll‐out in a “real‐world” setting to complement shared decision making with healthcare professionals. Otherwise, even if researchers develop a decision aid and show that it “works,” the opportunity for benefit will be lost if it is not used (Bauer & Kirchner, 2020). Prioritization of qualitative research data combined with the codesign ethos that underpinned the codevelopment of Lynch Choices™ has primed it for successful uptake into routine clinical practice. This maximizes the potential to improve health outcomes, reduce inequity of care, and empower Lynch syndrome carriers to make the decisions that are right for them, at the right time.

## AUTHOR CONTRIBUTIONS

KK was the lead researcher, wrote the original draft of the manuscript, edited, and prepared the final version. All co‐authors approved the final version to be published and agreed to be accountable for all aspects of the work in ensuring that all questions related to the accuracy or integrity of any part of the work were appropriately investigated and resolved. Conceptualization: KK, KM, LT, DE, CF. Investigation, data curation: KK. Methodology, analysis: KK, KM, DE, CF. Supervision: KM, DE, CF.

## FUNDING INFORMATION

Funding for this project was provided by Cancer Research UK Catalyst Award CanGene‐CanVar [C61296/A27223]. Preparation of this manuscript was supported by KK attending the University of Southampton Faculty of Medicine/Faculty of Environmental and Life Sciences writing retreat in January 2024. EJC is funded by a National Institute for Health and Care Research (NIHR) Advanced Fellowship (NIHR300650) and the NIHR Manchester Biomedical Research Centre (NIHR203308). HH/KK are part‐funded by Senior/Mid‐Career Research Fellowships at the NIHR Exeter Biomedical Research Centre (NIHR203320).

## CONFLICT OF INTEREST STATEMENT

The authors have no relevant conflicts of interest to disclose.

## ETHICS STATEMENT

Human studies and informed consent: Approval to conduct this human subjects research was obtained from the study sponsor, University of Southampton, the National Research Ethics Service and Health Research Authority (REC reference 22/NI/010, IRAS Project 312473). All procedures followed were in accordance with the ethical standards of the responsible committee on human experimentation (institutional and national) and with the Helsinki Declaration of 1975, as revised in 2000. Informed consent was obtained from individuals who completed the interviews.

Animal studies: No non‐human animal studies were carried about by the authors for this article.

## PATIENT CONSENT STATEMENT

All participants gave their informed consent prior to inclusion in the study.

## BEST TRAINEE PAPER AWARD STATEMENT

The research presented in this paper was conducted while the first author was fulfilling the degree requirement for a PhD in Health Sciences.

## Supporting information


Appendix S1:

## Data Availability

The data that support the findings of this study are available upon request from the corresponding author. The data are not publicly available due to privacy or ethical restrictions.

## Footnotes

^1^
The path_MMR nomenclature used in this manuscript matches the nomenclature used in the Prospective Lynch Syndromes Database and widely across Europe. This nomenclature is clear and gene‐specific, important due to the different cancer risks and penetrance estimates for carriers. Other nomenclature used internationally includes, for example, “MLH1 carrier” or “individual with a pathogenic variant.”
