# Supplementary material for: Optimizing risk‐reducing surgery and aspirin decision aids for Lynch syndrome carriers using the person‐based approach: A think‐aloud interview study
Source: J Genet Couns. 2025 Aug 7;34(4):e70089. doi: 10.1002/jgc4.70089 (PMC12329710; doi:10.1002/jgc4.70089)
Supplement: Supplementary file 1 — Appendix S1: [file JGC4-34-0-s001.pdf]

Supplementary File

Title:

Optimising a decision support intervention for people with Lynch syndrome using the Person-Based Approach: A think-aloud interview study

Authors:

Kelly Kohut[1,2], Kate Morton[1], Lesley Turner[3], Rebecca Foster[1], Elizabeth K Bancroft[4,5], John Burn[6], Emma Crosbie[7], Mev Domínguez Valentin[8], Mary Jane Esplen[9], Helen Hanson[10,11], Karen Hurley[12], Pål Moller[8], Neil Ryan[13], Katie Snape[2], on behalf of the CanGene-CanVar Patient Reference Panel, on behalf of the International Lynch Decision Aid Stakeholder Panel, Diana Eccles[14], Claire Foster[1]

[1] Centre for Psychosocial Research in Cancer: CentRIC, School of Health Sciences, University of Southampton, Southampton, UK
[2] South West Thames Centre for Genomics, Clinical Genetics Service, St George's University Hospitals NHS Foundation Trust, London, UK
[3] Patient and public collaborator
[4]Urology Genetics, The Royal Marsden NHS Foundation Trust, Downs Road, Sutton, SM2 5PT, UK
[5]Oncogenetics Team, The Institute of Cancer Research, 15 Cotswold Road, Sutton, SM2 5NG, UK
[6] Faculty of Medical Sciences, Newcastle University, Newcastle upon Tyne, NE1 7RU, UK
[7] Division of Cancer Sciences, University of Manchester, St Mary's Hospital, Oxford Road, Manchester M13 9WL, UK
[8] Tumour Biology Department, Institute for Cancer Research, Oslo University Hospital, Oslo, Norway
[9]Department of Psychiatry, Faculty of Medicine, University of Toronto, Toronto, Canada
[10]Peninsula Clinical Genetics Service, Royal Devon University Healthcare NHS Foundation Trust, Exeter, UK
[11]Department of Clinical and Biomedical Sciences, University of Exeter Medical School, Exeter, UK
[12]Stanford R Weiss, MD Center for Hereditary Colorectal Neoplasia, Cleveland Clinic, Cleveland, USA
[13] Department of Gynaecological Oncology, Royal Infirmary of Edinburgh, Edinburgh, UK
[14] Faculty of Medicine, University of Southampton, Southampton, UK

Contents

INVITATION LETTER 3

PATIENT INFORMATION SHEET 4

CONSENT FORM 7

COMMUNITY FLYER 9

THINK-ALOUD INTERVIEW GUIDE 10

SAMPLING QUESTIONS TO ASSESS DIVERSITY, INCLUSION AND HEALTH LITERACY 12

FIGURE S1. EXAMPLE OF THE PROTOTYPE DECISION AIDS BEFORE AND AFTER REFINEMENTS BASED ON THINK-ALOUD INTERVIEW DATA. 15

TABLE S1. TABLE OF CHANGES DOCUMENTING CANGENE-CANVAR PATIENT REFERENCE PANEL MEMBERS' FEEDBACK ABOUT THE STUDY DOCUMENTS, REVIEWED BEFORE THE STUDY ETHICS APPLICATION. 19

TABLE S2. ADDITIONAL EXAMPLES FROM TABLE OF CHANGES PRIORITISING POSSIBLE CHANGES TO THE PROTOTYPE DECISION AIDS BASED ON COMMENTS FROM THINK-ALOUD INTERVIEW PARTICIPANTS (N=20 LYNCH SYNDROME CARRIERS) 30

TABLE S3. ADDITIONAL QUOTES FROM THINK-ALOUD INTERVIEWS USED TO CONSTRUCT OVERARCHING THEMES AND SUBTHEMES TO INFORM OPTIMISATION OF LYNCH CHOICES TO OVERCOME BARRIERS TO ENGAGEMENT AND PROMOTE SHARED DECISION-MAKING. 59

Invitation letter

Would you like to improve support for people with Lynch syndrome?

Dear ______,

People with Lynch have told us that they would like to have better support, including up-to-date information that is easy to find.

Cancer Research UK have funded us to make a booklet and website to help people with Lynch.

I would really like to hear what you think about it so far.

Why have I been invited?

- We really value your opinion. You know best what is important to you.
- You do not need a scientific or medical background
- We will not be testing your knowledge or digital skills
- You would look at the website or booklet and tell me what you think
- You can choose to talk to me by telephone or video call.
- This will not take any longer than 1 hour
- We want to talk to people **from all backgrounds** and especially people who have not taken part in research before
- More information is attached for you to read
- We will offer a £30 Amazon voucher for your time

You can help create better information and support for people with Lynch and their families.
Thank you

Please click here or contact me if you would like to take part, or if you have any questions: [contact telephone number and email included]

Patient information sheet

Information about the Lynch choices research

What is the purpose of this research?

To make a website and booklet to help people with Lynch syndrome make decisions about their health. Lynch is a genetic condition that runs in families and causes an increased chance of getting some cancers in the future.

Who is doing the research?

My name is Kelly Kohut, and I am a Genetic Counsellor (someone who gives information about how genetic conditions might affect someone and their family).

Who is sponsoring and funding the research?

University of Southampton is sponsoring, and Cancer Research UK is funding the research.

Who can take part?

- People with Lynch syndrome aged18 years or older
- We expect 30 people to take part

What are the benefits of taking part?

- You might help others with Lynch and their families in the same situation
- It might help you think about your own decisions about Lynch

What does the interview involve?

- A conversation with me (Kelly Kohut) lasting no longer than 1 hour
- I would first like to talk to you about your experience of having Lynch
- You will then look at the website or booklet and tell me what you think

- You can choose to talk to me by telephone or video call. With your consent, I will record this (sound only), so we can capture your views correctly.
- There will be some questions about your gender, age, ethnicity, whether you have had cancer, and how easy you find it to understand medical information
- Taking part will not affect your medical care
- We will offer a £30 Amazon voucher for your time

Are there any risks involved?

- Some people may feel upset talking about their chance of getting cancer in the future and making choices about how to manage this
- You can choose not to answer any questions if you do not feel comfortable
- I will not be able to answer personal questions about your care. I may suggest you speak to your GP, NHS 111, patient groups or charities.

What will happen to my personal information?

- We will type up the conversation from the recording and keep it for 10 years. These records will not include your name or details to identify you.
- The records will be stored on a secure computer at the University of Southampton. Only the research team can see the records.
- When the research is complete the recordings will be deleted
- Your name and contact details will be stored during the research so we can contact you, and then deleted
- You can see here to read more about your personal information:

Data Protection Privacy Notice

The University of Southampton conducts research to the highest standards of research integrity. As a publicly-funded organisation, the University has to ensure that it is in the public interest when we use personally-identifiable information about people who have agreed to take part in research. This means that when you agree to take part in a research study, we will use information about you in the ways needed, and for the purposes specified, to conduct and complete the research project. Under data protection law, 'Personal data' means any information that relates to and is capable of identifying a living individual. The University's data protection policy governing the use of personal data by the University can be found on its website (https://www.southampton.ac.uk/legalservices/what-we-do/data-protection-and-foi.page).

This Participant Information Sheet tells you what data will be collected for this project and whether this includes any personal data. Please ask the research team if you have any questions or are unclear what data is being collected about you.

Our privacy notice for research participants provides more information on how the University of Southampton collects and uses your personal data when you take part in one of our research projects and can be found at http://www.southampton.ac.uk/assets/sharepoint/intranet/ls/Public/Research%20and%20Integrity%20Privacy%20Notice/Privacy%20Notice%20for%20Research%20Participants.pdf

Any personal data we collect in this study will be used only for the purposes of carrying out our research and will be handled according to the University's policies in line with data protection law. If any personal data is used from which you can be identified directly, it will not be disclosed to anyone else without your consent unless the University of Southampton is required by law to disclose it.

Data protection law requires us to have a valid legal reason ('lawful basis') to process and use your Personal data. The lawful basis for processing personal information in this research study is for the performance of a task carried out in the public interest. Personal data collected for research will not be used for any other purpose.

For the purposes of data protection law, the University of Southampton is the 'Data Controller' for this study, which means that we are responsible for looking after your information and using it properly. The University of Southampton (and NHS sites if you were recruited from an NHS Trust) will keep identifiable information about you for after the study has finished after which time any link between you and your information will be removed.

To safeguard your rights, we will use the minimum personal data necessary to achieve our research study objectives. Your data protection rights – such as to access, change, or transfer such information - may be limited, however, in order for the research output to be reliable and accurate. The University will not do anything with your personal data that you would not reasonably expect.

If you have any questions about how your personal data is used, or wish to exercise any of your rights, please consult the University's data protection webpage (https://www.southampton.ac.uk/legalservices/what-we-do/data-protection-and-foi.page) where you can make a request using our online form. If you need further assistance, please contact the University's Data Protection Officer (data.protection@soton.ac.uk).

What happens if I change my mind?

- You can change your mind and stop taking part at any time without giving a reason
- You can choose not to answer any questions if you do not feel comfortable
- If you tell me you would like to stop the conversation, all information collected so far will be deleted
- If you contact the research team after the conversation to stop taking part, we will not contact you again, but the typed record will still be used for the research. This will not include your name or details to identify you.

What happens if there is a problem?

- If you have a concern about any part of this research, you should speak to the researchers who will do their best to answer your questions
- If you remain unhappy or have a complaint, please contact the University of Southampton Research Integrity and Governance Manager [contact email and telephone number provided]
- Complaints in the NHS can be directed by Patient Advice and Liaison Service (PALS). [What is PALS (Patient Advice and Liaison Service)? - NHS (www.nhs.uk)]

Contact details

For more information or questions, please contact [contact email and telephone number provided]

Consent form

Consent form for the Lynch choices interview

Please tick the box(es) if you agree with the statement(s):

• I have read the information (Participant Information Sheet_Interview_04-03-2022_v1)	
• I have had the chance to ask questions	
• I understand what I will be doing	
• I understand that I do not have to take part and that I can stop taking part at any time without giving a reason	
• I am happy for the researcher to record our conversation	

• I understand that what I say will be typed up. It will have a code name so that I cannot be identified.	
• I understand that information I give will be looked at by researchers from the University of Southampton. If they are worried about my safety or someone else's, they may need to share my information with support services.	
• I am happy for things I say to be quoted in reports or presentations about the research. I will not be personally identified.	
• I agree to take part	

Type your name ……………………………………………………………………………….

Contact details ……………………………………………………………………………….

Date (completed automatically by the programme) . ………………….

Click <u>next</u> to complete the form

(next page states: Thank you for completing this consent form. A researcher from the University of Southampton will be in touch with you soon.)

The researcher will add their name to this section of the consent form before the interview and provide a copy to the participant:

Name of researcher……………………………………………………………………………

Date……………………………………………………………

Community flyer

Do you have Lynch syndrome?

Would you like to improve support for people with Lynch?

People with Lynch have told us that they would like to have better support, including up-to-date information that is easy to find.

Cancer Research UK have funded us to make a booklet and website to help people with Lynch.

My name is Kelly Kohut. I am a researcher.

I would really like to hear what you think about it so far.

What is involved?

- You would look at the website or booklet and tell me what you think
- You can choose to speak to me by telephone or video call
- This will take no longer than 1 hour
- We really value your opinion. You know best what is important to you.
- We want to talk to people **from all backgrounds** and especially people who have not taken part in research before
- We will offer a £30 Amazon voucher for your time

You can help create better information and support for people with Lynch and their families. Thank you.

Please feel welcome to share this leaflet with your family members.

Please click here or contact me if you would like to take part, or if you have any questions: [contact email and telephone number provided]

Think-aloud interview guide

Introduction: *[see Introductory Interview schedule_20220304_v1]*

If the participant has chosen to split the introductory and think-aloud interviews to have them on different days, then a brief re-introduction and rapport-building/contracting session will be included (*in italics below*). If the think-aloud interview directly follows the introductory interview, this will not be needed.

- *Thank you for meeting with me last time. As you will recall, I am a Genetic Counsellor working on a Doctorate degree. My research involves working directly with patients and other experts to make a website and booklet to help people with Lynch syndrome make choices to manage their increased chance of getting cancer in the future*
- *How are you today? How have you been since our last meeting/call? Did our discussion make you think of any questions you would like to ask me about the research?*
- *Just to remind you, the purpose of our conversation today is to talk about what you think of the website and booklet so we can make it better and make sure it meets people's needs*
- *Do you have any questions before we start?*

Think-aloud interview:

- Thank you for completing the consent form. Are you still happy to take part? Do you have any questions about the consent form?
- Our conversation should last from 30 minutes to no more than 1 hour. This will be up to you, depending on how much you have to say. We will offer a £30 Amazon voucher at the end to thank you for your time.
- There are no right or wrong answers, so please say any thoughts which spring to mind, even if you think they might not be important. Your thoughts about the Lynch website/booklet are really important to us, so the more you can tell me about it the better.
- Please do feel free to say any negative thoughts you may have about the website/booklet as these will be useful in helping us to make it better. Please feel comfortable being honest.
- This might be different from surveys or questionnaires that you have done before, as I really want to hear as much as you have to say. I will leave some pauses so you can have time to think as you're talking, and so you can tell me in detail what it is like for you reading and looking at the information, but I want to reassure you that I am always listening.
- If you have any questions while we are going through, I will be very happy to answer them but it's probably best if I answer them at the end.
- If you are happy, I will record the conversation, so I can listen again to what was said and make sure I can record your views correctly. You will not be identified personally in any papers or presentations about the research.

- We can take a break at any time you like. Please just let know and I can pause the recording, or I can always call back to finish the conversation another time if that is better for you.
- You can choose not to take part at any time. If you change your mind about taking part during the conversation, we can delete all the information you have given us so far. If you change your mind in the future after we've finished our conversation, we will not contact you again, but we would still use the information you gave us.
- Is there anything you would like to ask me before we start?
- If you are happy, I will start recording now.

<u>Think-aloud questions</u>

What are your first thoughts about this page/information/activity?

What are you thinking about now?

Can you tell me a bit more about why you think that?

Can you tell me what it is that you like/don't like about that?

<u>After they have finished viewing:</u>

Overall, how do you feel about the Lynch choices website/booklet?

Can you tell me about anything you felt was good about the website/booklet?

Can you tell me anything about the website/booklet that you were less keen on or felt was less relevant to you?

What do you think should be changed?

How would you feel about using the Lynch choices website/booklet?

Would you have any concerns about using the website/booklet?

Which parts of the website/booklet would you recommend to other people?

Can you tell me about anything else that you think might be useful for you when you are thinking about making decisions related to Lynch?

Is there anything else you would like to talk about that we haven't already discussed?

Sampling questions to assess diversity, inclusion and health literacy

These questions will be asked at the end of the introductory interviews. They will be included as optional questions in the survey. Additional questions may be added to explore specific issues as they emerge and/or ensure inclusive and diverse recruitment.

A bit about you:

We would like a little bit of information about you. This is so we can make sure the decision aid can help everyone who needs it.
We keep this information **secure and confidential.**
All questions are **completely optional to answer**. You can leave them blank if you prefer.

1. **Where did you hear about the Lynch choices website and booklet?**

a. Genetics service
b. Cancer/oncology service
c. GP
d. Lynch Syndrome UK
e. Bowel Cancer UK
f. A friend or family member
g. Other (please state here): ________________________

We may add additional or remove options from here, as our dissemination strategy changes

2. **How old are you?**

- 18 to 25
- 26 to 40
- 41 to 60
- 61 to 70
- Over 70

3. **When did you leave full time education? (if you are still in education, please select the stage you are at now**)

- Before finishing school
- After finishing school
- After finishing university
- After postgraduate studies

4. **What is your gender identity?**

☐Male

☐Female

☐ Other/ Prefer to self-describe: (optional to add text):____________________

☐Prefer not to say

5. **What is your ethnic group?**

  - Black Caribbean
  - Black African
  - Black Other
  - Bangladeshi
  - Chinese/ South East
  - Indian
  - Pakistani

- White British
- Any other White
- Asian
- Other: ____
- Prefer not to say

6. **Have you ever been diagnosed with cancer?**
   - No
   - Bowel (colon) cancer
   - Endometrial (womb/uterus) cancer
   - Other (please state here): ________________________________

7. **What is your postcode (or country if outside the UK)?** This is not to identify you, but so we can see if we have included people's opinions from across the UK and any other countries.
   UK post code___________________

Other country___________________

8. **Do you have any access requirements you would like me to be aware of?**
   Yes
   If yes, please tell me more____________________________________
   No

Finally, we have 3 short questions we would like to ask you about how easy you find it to understand health information (adapted from [1]).

9. **How often does someone (like a family member, friend, hospital/clinic worker, or caregiver) help you read hospital materials?**

(1) Always
(2) Often
(3) Sometimes

(4) Occasionally

(5) Never **_Answer:_**

---

10. How confident are you filling out medical forms by yourself?

(1) Extremely
(2) Quite a bit
(3) Somewhat
(4) A little bit

(5) Not at all **_Answer:_**

---

11. **How often do you have problems because you find it difficult to understand written information about medical conditions?**

(1) Always
(2) Often
(3) Sometimes
(4) Occasionally

(5) Never **_Answer:_**

Figure S1. Example of the prototype decision aids before (a) and after (b) refinements based on think-aloud interview data.

1a)

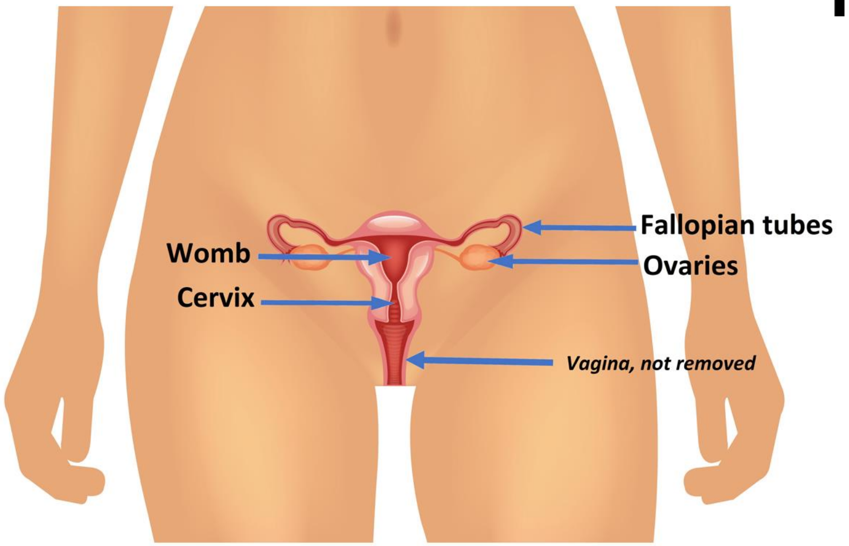
Womb
Cervix
Fallopian tubes
Ovaries
Vagina, not removed

1b)

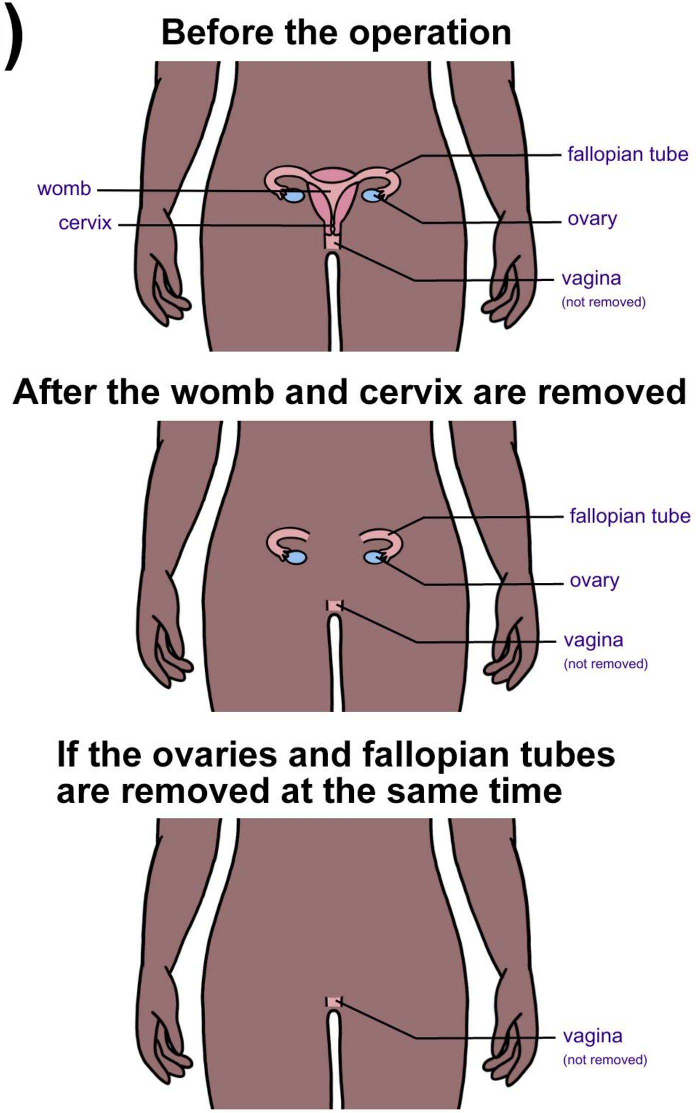
Before the operation
womb
cervix
fallopian tube
ovary
vagina
(not removed)
After the womb and cervix are removed
fallopian tube
ovary
vagina
(not removed)
If the ovaries and fallopian tubes are removed at the same time
vagina
(not removed)

2a) What are the chances of getting womb cancer and ovarian cancer?

- Some people like to know how likely they are to get cancer, to help them decide about having an operation.
- These images show the average number of people who get womb or ovarian cancer in the UK by the age of 75, with and without Lynch syndrome.
- These numbers are for people who have not had their womb or ovaries removed.

	Number who develop womb cancer in their lifetime	Number who develop ovarian cancer in their lifetime
People with a MLH1 gene alteration, Lynch syndrome	Between 3 and 4 out of 10 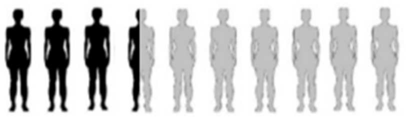	Just over 1 out of every 10 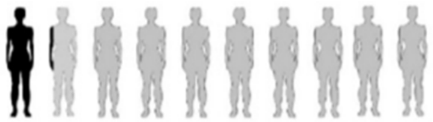
People in the general population without womb or ovaries removed	Fewer than 1 out of every 10 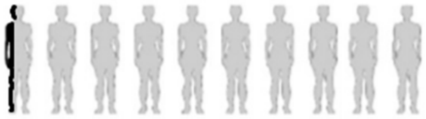	Fewer than 1 out of every 10 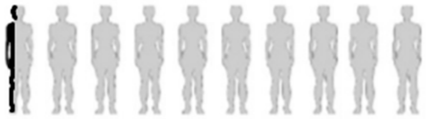

2b)

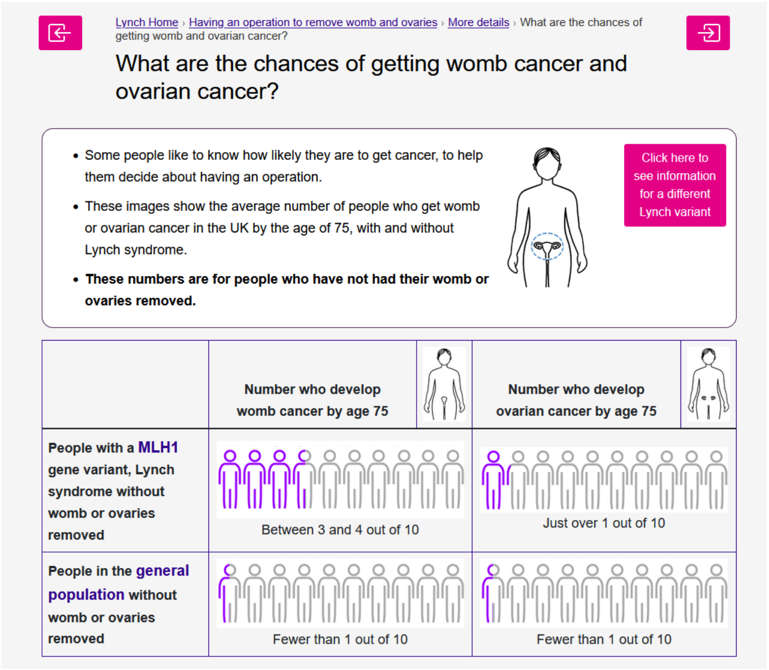

3a)

Number who develop bowel cancer in 10 years

Just under 1 out of every 10 (0.7)

People with Lynch syndrome who took aspirin

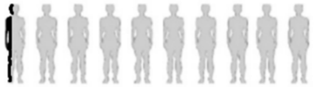

Just over 1 out of every 10 (1.3)

People with Lynch syndrome who did not take aspirin

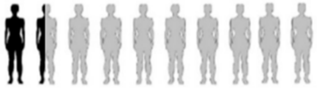

You can read more about the CaPP2 study here if you would like. https://www.capp3.org/about/trial-history.aspx

3b)

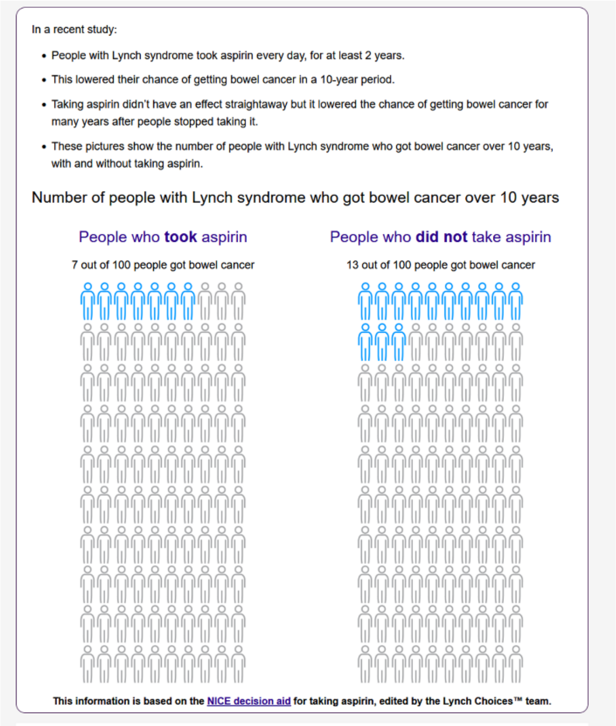
In a recent study:

- People with Lynch syndrome took aspirin every day, for at least 2 years.
- This lowered their chance of getting bowel cancer in a 10-year period.
- Taking aspirin didn't have an effect straightaway but it lowered the chance of getting bowel cancer for many years after people stopped taking it.
- These pictures show the number of people with Lynch syndrome who got bowel cancer over 10 years, with and without taking aspirin.

Number of people with Lynch syndrome who got bowel cancer over 10 years

People who **took** aspirin

7 out of 100 people got bowel cancer

People who **did not** take aspirin

13 out of 100 people got bowel cancer

This information is based on the NICE decision aid for taking aspirin, edited by the Lynch Choices™ team.

Legend: Before (a) and after (b) refinements based on think-aloud interview data. 1a) image of gynaecological organs, refined in 1b) to reflect diversity in skin tone and variation in types of hysterectomy +/- removal of ovaries. 2a) icon arrays showing risk reduction from hysterectomy, refined in 2b) to differentiate womb and ovarian cancer risk, with pictures of organs and more striking use of colour for icons. 3a) visualisation of risk reduction from taking daily aspirin, refined in 3b) to include background of CaPP2 study and more striking colours in icon array with gender-neutral outline.

Table S1. Table of Changes documenting CanGene-CanVar Patient Reference Panel members' feedback about the study documents, reviewed before the study ethics application.

**Document reviewed by Patient Reference Panel before ethics application for think-aloud interview study**	**Negative Comments**	**Neutral comments**	**Positive Comments**	**Possible change**	**MoScoW**	**Implemented?**
Invitation to interview	Do you need to say more re: what kind of support? What do you mean?			explain what support (trusted, up-to-date info that is easy to find)	Must	yes
Invitation to interview			I like that you don't use the word 'decision aid'. I wouldn't have a clue what that is.	none needed (but consider changing other documents that mention 'decision aid')	NA	NA
Invitation to interview		That first sentence, maybe swap it around a bit…people with Lynch syndrome have told us…		swap order of words to make it clearer	Should	yes
Invitation to interview	a) You don't need any special skills – put that first, some people may	a) Do you say 'special skills' because it's a website, do you	I like 'we really value your opinion as you	lots of discussion, incorporate suggested changes	Must	yes

	not realise<br>b) I don’t know about ‘special skills’ – is it that you don’t need to have a scientific background ? Saying we really value your opinion is good.<br>c) could take that out completely then. Just say we really value your opinion, we want to know what’s important to you<br>d) Not sure about the word ‘expert’ – if you’re not confident in what you know, may feel like you’re not an expert. But like the idea of saying that you know best. People feel like they don’t know it all and that’s exactly who we want to target.	mean digital rather than medical?<br>b) You have to be careful using the word ‘special’<br>c) Do you want to say about being IT savvy or computer savvy, maybe clarify that? Seen other PIS that say you don’t need scientific or medical background. What patients do have is an understanding of their condition.<br>d) Seen it worded like ‘this won’t be about testing your knowledge or digital skills, we’re testing our product – you’re here to test us’<br>e) could be more about hearing what’s important to you	are the expert in your condition’.			
Invitation to interview	put the £30 voucher thing at the end			move to last bullet point	Could	yes
Invitation to interview	a) esp people who have not taken part before. Felt we didn’t need to say people of	need to include digital literacy too, learn most from		Inlcude all by ‘we really value your opinion as you are the expert in your condition’.	Could	yes

	all backgrounds, you've only got one background. That comes from who you're targeting.<br>b) I think I've seen it overcome by working directly with those communities, the people who aren't engaging with research.	people who have low digital confidence.				
Invitation to interview		It says interview could take up to an hour – maybe say interview should not take any longer than one hour.		clarify no longer than one hour	Should	yes
Participant information sheet for interview	You've got to move Cangene-Canvar, no one has any idea what that means and that's the first thing you see. Everyone has heard of Cancer Research UK; need to change the order round.			remove from all documents and add CGCV website to footer instead	Must	yes
Participant information sheet for interview	Don't call it a 'decision aid'. No one knows what that is. What you are developing is a website. Group agreed.			remove words decision aid from all documents and just call it a website/booklet	Must	yes

Participant information sheet for interview	Aspect about the benefit of taking part in the research, take out that point that you may not get anything, if you start off by talking about helping families in the same situation, I get joy from thinking about I can help others, that's why I'm on things like this.	b)		change as suggested	Must	yes
Participant information sheet for interview		a) what about speaking to family members, my wife would have loved to found this?<br>b) it would be useful for people before they get tested for Lynch, if a family member has been diagnosed.		mention in ethics applicationt that we might include carers/relatives/pre-test but the intial interviews will be reserved for people with Lynch due to limited resource (time and funding)	should	yes
Participant information sheet for interview	a) this one and appendix A – mentioned that you might meet in person, just wondered what incentive would be for people – travel, inconvenience…<br>b) do you need			take out 'in person' and mention in ethics application can do for local patients if requested/preferred	should	yes

	something re precautions you would take? Would it be useful to make it plain that you don’t have to do it in person, it can all be virtual. It might put people off.<br>c) it doesn’t say where in person, could be Southampton or Glasgow. I think you should take that ‘in person’ out.					
Participant information sheet for interview		Do you need to inform the participants how you will feed back to them the value of their taking part in this? And you’ve not put in there that you might use this at research and conferences, to let them know how their data will be used.	I like the first person approach.<br>Having privacy notice and data protection as optional link to click is good.	Consider communications to share the outputs with participants once data has been analysed and reported	should	consider in future
Participant information sheet for interview	You can look at it more another time.' What does that mean? If I’ve had enough after 20mins can I			Take this out and ask them at the end of the interview if theyre happy to be contacted again to look at more parts of the website	Should	yes

	break off and come back, or can I be involved in more research at a later date?					
Participant information sheet for interview		Change the question 'I will ask a bit about you having Lynch' to 'I would like to talk to you about your personal experience of having Lynch so we can make the website better'.		change as suggested	Should	yes
Participant information sheet for interview	'This is to make sure we speak to a diverse range of people, you do not have to answer'. Take that bit out, it goes without saying.			Take out as suggested	Should	yes
Participant information sheet for interview		a) Maybe you need an extra line to say if you're not comfortable to answer any questions, you don't have to. It needs to be in there, but it applies to all the interview not just the demographic questions.		suggested moving it to come under the section about risks involved – PRP agreed it does flow on from there	Should	yes

		b) That could possibly go in the bit about what happens if I change my mind, that might be a natural place to put that. If you just give more details, if you feel upset at any point, you're free to stop.				
Community flyer and text for invitation for interview	Same discussion as above re: logo and decision aid			as before, remove CanGene-CanVar logo and put website in footer, remove reference to 'decision aid'	Must	yes
Community flyer and text for invitation for interview	a) I think take out the picture, it doesn't add anything, potentially isolates some of your audience<br>b) It might be useful if you're doing targeted recruitment to select a picture relevant to each target group.<br>c) Problem with changing people, it makes a lot of work. Could have a medical picture. You can never get people to suit every possible group<br>d) I find scientific	a) If you're having an image, you should have a woman on there, otherwise it makes it look like you only want to hear from men. I like the fact you have different generations, but have one with a woman in.<br>b) What would you use if not people? Cool picture of a gene?<br>c) if you're not science based and	a) I disagree, visual person, like to see a picture.<br>B) images – people like to see an image on a PIS or booklet, maybe something like an infographic would be better than a photo, symbolic of diversity and inclusion.	Remove reference to researcher being a Genetic Counsellor. Change image to more diverse group of people including women.	Must	yes

	pictures off-putting, and I think you said in both you're a genetic counsellor – don't explain what that is here. Anyone who hasn't got that experience has no idea what that is. People might not feel comfortable sending to a mate.<br>e) Kelly: Guidance in ethics said you should explain who you are and what your title is.<br>PRP: In this context, you're a researcher. Just say that.	see a gene. People might write it off, don't even understand the picture.				
Community flyer and text for invitation for interview		You asked, how do we disseminate this to people who don't engage wtih social media – would it be helpful to ask people to share this with affected members of their family who may not be engaged in social media? A lot of people on LSUK are spouse or daughter of another person		Add a line saying, 'You are welcome to share this leaflet with your family members'	Should	yes

		with Lynch who isn't on there.				
Introductory interview guide		RE: introductory bit, would it be easier to understand if you talk about genes that can increase your chance of getting cancer? Call it surgery that could reduce your risk. I wonder if it's relevant to have that bit about adapting for other genes. In the second para – I'd be more positive, get rid of the 'and try' to make it better.		make changes as suggested	Should	
Introductory interview guide	Get rid of tape recording – just recording.			remove as suggested	Should	yes
Introductory interview guide	a) The word 'risk' - we've been having lots of conversations about – risk sounds worse than chance. Chance is preferred - chance of getting cancer rather than cancer risk. People look on it more favourably	You say 'and make sure I can record your views correctly', is that ok? I don't think that needs to be in there. You say that again further down, 'is that ok'.		removed as suggested	Should	yes

	b) your risk of getting cancer in future, sounds better than 'your cancer risks'. Be a little more directive.					
Introductory interview guide	The question about 'what do you think affects your decisions about cancer risks' – I don't like the wording of that. Almost too open – might need a bit of work. Didn't ring true to me.	More like 'what sort of things come in to play when making decisions about cancer risks'		reworded	Must	yes
Introductory interview guide	Even not use the word 'trust' just say 'what sources of info have you found helpful'? Don't like the word trust, it's an awkward one. Agree, GP is not the expert, just the gatekeeper.	a) question re: how much you trust the information from your HCP. Just wanted to ask about those questions<br>b) why do you want to know about trust?<br>c) I don't trust the GP but I trust the oncologist and surgeon<br>d) any sources of info that you trust more - need to tweak that question, clarify.<br>e) would it be interesting to know	I think there is something about trust though, if we want to make this something that everyone would use, especially people who have barriers to access healthcare, trust is huge, we know that groups who don't normally take part in research don't trust the NHS have poorer health outcomes, more likely to trust community leaders. Who you trust	reworded	Must	yes

		how they got signposted to that person they trust?	determines on what treatment you get. I believe that trust is really important thing to understand about this and how you label it, for some people labelling it an academic institution will create greater trust than labelling it a health institution.			
Introductory interview guide		Is it worth asking if theyre participating in any other studies, like CaPP studies? Helps to see if they are novice to research.		added a question about any other research studies	Should	yes
Introductory interview guide		Do we need to ask how things have changed during COVID? Many people haven't had the support of HCPs, maybe more reliance on non-approved sources.		added question	Should	yes
Introductory interview guide	3 literacy questions, can they be modified? Two of them sound quite clumsy. 'How often do you have			reworded as suggested and changed to 'adapted from [ref Chew et al.]'	Should	yes

	someone help you read materials?' Could it be 'How often does someone…' And could it be 'How often do you have problems because you find it difficult to understand written information?' Make it active, not passive.					

Table S2. Additional examples from Table of Changes prioritising possible changes to the prototype decision aids based on comments from think-aloud interview participants (n=20 Lynch syndrome carriers)

Legend: The first half of participants (n=10, participant ID numbers p001-p010) looked at the prototype hysterectomy decision aid, and the second half (n=10, participant ID numbers p011-p020) looked at the prototype aspirin decision aid. The prototypes were iterative refined after the first two interviews, then after each batch of five interviews to optimise the decision aid before gathering more participant feedback.

Participant ID	Negative comments	Neutral comments	Positive comments	Possible change	MoSCoW prioritisation
p001	I didn't realise, depending on which type of Lynch, you could have just your womb out or they take the ovaries.		I love that it exists. I think the way it's presented is helpful. It's useful. I like it.	Add on first page that ovaries are sometimes not removed, depending on which Lynch gene is involved.<br><br>Ask for Lynch alteration from the start so that only people with PMS2 see info about ovaries not being removed, to prevent confusion.	Must have<br><br>Would have
p001	The other thing I think that's missing, or not clear, so I hadn't seen a Gynaecologist before and I found that my genetic		I like the way the information is presented here.	Change to: 'Your GP or genetics service could help to refer you if you have not seen a gynaecologist' with hover over	Must have

	counsellor, she was like, go see your GP and I kept getting stuck in this loop like. So yeah, I'm not sure if that's the case for every patient. But that for me was, was just a loop.			definitions. *make changes throughout the website	
p001	I really hate that icon, because to me that symbol always means, like, I know you're sick, but I think it's a bit harsh. [Interviewer: Could you say more?] I think it's just like a big red thing in the middle, but you just found out you've got Lynch. You've got to go figure out whether you're going to get yourself into early menopause, or not, in my case. I just feel like it's like you're stopping it, yet you're stopping yourself having surgery and it's like, obviously I would want to do anything. I want to do anything to reduce my chance of cancer, but then also, I want to have a kid, so? I just feel like it’s a bit harsh.			Change icon, use version without the red. Add illustrations of person with womb & ovaries in situ or removed.	Could have
p001	Why is it saying 'Don't have the operation'? So that's little help. It's just the fact that it says ‘do not’. It's the only thing I don’t like. ‘Cause, it’s like somebody is telling me ‘do not have the operation’. Rather than not have the operation because you were happy. Like if my mom said don't have the operation or something. Like than to not have the operation. [It's] the wording, yeah. But otherwise fine.			Change headings to 'If you choose to have the operation', 'If you choose not to have the operation' and 'If you choose to wait to decide'	Must have

p001	So I would say that this text needs to be past the picture. I think the picture's just too big. I think it's more important to have the text, but yeah, the picture doesn't do anything for me and I think it's really important information about having future children and things like that. It kind of just makes it feel a bit unimportant by having the big picture in the middle.		I like this. [Prompted: What do you like about it?] It's easy to read, specific to *MLH1,* comparing to the average population, so you can see how much more is by having Lynch. And it's split between womb and ovarian. That's important.	Make picture smaller and put below all the text in the first box.	Should have
p001	I wonder why each in the text it's like 40/50/60. I wonder if you can show the percentage as well. Stick basically next to it. [Interviewer: So you like to see the percentage as well as the 2 out of 100?] Yeah, it's just easier having the percentage as well if possible.	Sorry, is this is just womb? [needed to scroll to see ovarian] I feel like maybe consolidating the bullets, could be done in a more succinct way. But I like the graphic.	I like how the information I presented. [Interviewer: Is there anything you don't like about it?] Um. No, I like it.	Move to be side by side with the icon arrays: 'This shows that out of 100 women, 2 out of 100 have had womb cancer by age 40, 13 out of 100 have had womb cancer by age 50, 28 out of 100 have had womb cancer by age 60.' Separate drop-downs for 'How does my chance of getting womb and ovarian cancer change with age?' Add illustrations of womb and ovaries from first page for visual reminder.	Must have
p001	[about menopausal side effects] All of them!			Anxious about getting all the side effects of the menopause, need to reassure people.	Must have

				Explain that these are some common symptoms of the menopause. Most people only experience some symptoms, not all of them. Instructions to continue to see about HRT.	
p001	I’m trying to see, where do we try and educate people that surgical menopause is different to natural menopause? Would they know that already?			Bold this sentence on the menopause page: 'It is usually a gradual process that happens around age 45-55 years. Having your ovaries removed in an operation brings on menopause more suddenly.'	Could have
p002	Or wait to decide later, good to put that in. However, why would you wait in a life threatening condition? I'm probably reading too much into it, but the options are there and what they mean for you if you don't take it. So I take it if you were to wait to decide later, does something, does that open up to give any further information?			Add on the first page where it lists the options: 'Wait to decide later if you're not ready now'.	Should have
p002	Talk about endometrial. The Beginner's Guide to Lynch gives that from 50 to 80 and the percentage is from 10% to 45%. [Interviewer: so you find it helpful to have it broken down a bit more by age?] I think so. If I didn't have that to refer back to then just 4 out of 10 in the lifetime. Some people might live to 50 or 60 might not get to 80, but for the people who will live that bit longer, have that longevity. It probably is a bit more reassurance to look at it from that point of view.	[about box stating not possible to know…] Yeah. Well, you can't tell when people with Lynch syndrome will, will ever or	I like it with the graphics of the human body.	Add link to The Royal Marsden Beginner's Guide to Lynch Syndrome. Include lifetime risk to age 75.	Should have

		never develop cancer, that's right.			
p002	I know it sounds petty, but the colours could be a bit more vibrant. I think more vibrant the colours that stands out more. I like the blue and the lime green, but I think the pink needs to be like a, a shocking pink. So it's, it stands out. [Interviewers: What about the grey for people not expected to develop cancer?]: Yeah, that's fine. I just think the colours pop out the stronger they are. They pop out.			Make the pink darker.<br>We already include risk compared with general population on the main page. But need to include here too, broken down by age.	Could have
p002	So is there, is there any connection to Lynch syndrome [referring to Lynch Syndrome UK patient charity] on the page as well? I know it's your booklet, but is there any reference to that as well?		Fantastic. I think, unless you've got IT skills, it's great that you can do your research, but for people out there who have nothing or no one, this will be a lifeline to so many people.	Add Lynch Syndrome UK and Lynch Syndrome Ireland to support resources.	Must have
p003	Having the operation also makes you feel lots of other things, not necessarily related to cancer. Maybe it should be 'How does this make me feel about my cancer risk 'or something like that? Because it's, it feels like that's a bit of a broad statement perhaps because it's not just about the feelings about, I dunno what you could say instead… Active health, family. All of those things.		[about options grid lay-out]: I think that's really clear.	Change to 'How might this make me feel about my chance of cancer?'	Could have

p003	When you're given those sort of statistics, that's really powerful. I dunno if you could make that bit more 'cause that's the sort of thing I found difficult is like when things are really embedded in a website. You click, it's almost like you've stumble across them as opposed to... Maybe that should be pulled out and put more centrally somehow.		It's clever. If you click on bowel, presumably that would change. That's really clever. That's why I had my surgery. [extended colectomy for bowel cancer]	Include description of the linkout, describing the Prospective Lynch Syndrome Database (PLSD) website.	Should have
p003	It might be worth saying something, especially with a tablet about disadvantages. If you've got a compromised bowel absorption then obviously you're not gonna absorb it in the same way. I can't think of a time where it would be more appropriate to take a tablet. Because I think that's where my big problems occurred was when they just gave me what they give every old lady basically. And for me specifically, it wasn't. I guess it's probably cheaper maybe if I'm being very cynical.<br>So you're supposed to, um, rub [gel] on the top of your arm. So it's supposed to go sort of here or in inner thigh.		That's really good. Vaginal oestrogen as well. That's a good picture of that I think.	Explain that tablets are less often prescribed now because of the chance of blood clots. Note that people who have had bowel surgery may not be able to take tablets because of problems with absorption in the body. Change picture of gel showing where it is applied. Checked with expert stakeholders.	Must have
p004	It would be really important to link to other external sites, like NHS. Probably one of the biggest reasons apart from having a family, why I am not choosing to have this yet is 'cause I really am not looking forward to menopause. And I know that it has horrible side effects from what I've heard. And so that, that would			Link to sites like NHS. Remove: 'A side effect of the menopause is that your bones may become thinner over time, which makes them more likely to break.' Add: 'You may have some side effects of the menopause. However taking Hormone Replacement Therapy (HRT) is a good way to keep healthy	Must have

	be a huge decision-maker for me. Like I would almost be like, should I just like live with my risk of cancer as opposed to bringing this about early? Like that's a big thing, I think.			and feel well after having your ovaries removed. You can read more about this on the next page.'	
p004	This might answer some of the questions that I was just talking about. Like what are the chances of womb cancer, information about the menopause? So it sounds like actually a lot of the things I asked about are there.		I think that's clear so far.	Supports the idea that we need to let people know this is coming before they see the option grid table. Manage expectations that it is only a summary.	Should have
p004	I don't even know. I don't even have my gene there, so that would stump me. Like I feel like mine is none of those. Like, I feel like if you were to, if mine was in the list, I'd recognise it, but it's not any of those. I don't.			Add above the box to select your variant: 'If you do not know which Lynch alteration you have, you can click next and the website will show you information for all four Lynch variants.' If you want to see information that is specific to you, you can ask your GP or genetics service which alteration you have.'	Must have
p004	So question about support. It's mentioned speaking to a gynaecologist. I've never spoken to a gynaecologist in my life. I have no idea how to go about being referred apart from ringing my GP and I'm not gonna lie. It's impossible to get an appointment. I've gone to private healthcare because I cannot speak to a GP. If there are other people like me that have moved since their diagnosis, they might be in the same place. I don't even know who my genetics service are. I haven't spoken to them in 15 years. Now I live in a		I think that's really powerful. It's kind of, it's kind of scary actually. Um, I mean, no, I knew the risks were higher obviously than the general population, but it's literally just like putting it into this graphic, you know, with people, that, that really hammers it home. So I think that's	Change all sections mentioning GP or gynaecologist to "Your GP or genetics service can refer you to a gynaecologist, if you have not seen one before. Hover over 'genetics service' to see: 'If you're not sure who your genetics service is, you can ask your GP to refer you for an appointment with them.' 'You can click here to see where your local genetics centre would be, so you know what to expect.'	Must have

	complete different part of the country, I don't know who my local genetic service would be. I'm probably not like on that books. I've never spoken to them. So I, I don't even know how I would do that.		really powerful. Yeah. I think that's great.		
p004	It's a very small tactical point, but I just noticed there's nothing about breastfeeding and I'm sure that I've read somewhere that length of breastfeeding duration helps the chances. So I'm just wondering if that should be there as well.			Add: "If you want to read more about what affects your chance of ovarian cancer, you can click here". Link to Target Ovarian Cancer.	Should have
p004	My first initial reaction to this is, most people get these symptoms on a daily basis, like IBS or various other kind of things. Do you know what I mean? I know so many people that are like, oh, I'm so bloated today. I ate bread. A lot of people might read this and go, oh my God, I've got cancer. That might not be the case. I mean, I would read that and be like, wow, I have all of those things.			Add: 'Many people may find these symptoms are normal for them due to other reasons. The important thing is to watch out for any changes that are not normal for you, or do not go away.'	Must have
p004	The only slight thing is that I would have separate line for benefits and disadvantages.			Create separate rows for benefits and disadvantages.	Must have
p005	These are the chance, these are the risks that any woman would have, or anybody with a womb would have. It's not because you have Lynch syndrome, you have the risk of having these other problems. So just say fibroids, I dunno. It sounds like we are trying to convince you to do that because if you don't do that, you might get an ovarian cyst, but that's a risk			Add: 'Everyone with a womb and ovaries has a chance of some health problems, not related to cancer. These include fibroids and ovarian cysts.'	Should have

	everybody has, I don’t know, maybe I would say like any other person with ovaries, you still have the risk of, I don't know. Sounds a bit more neutral.				
p005	When you say Lynch syndrome without one’s ovaries removed. Maybe it complicates a little, gets a bit confusing. I'm finding this extremely helpful. Extremely good. I have to say, apart from these very small bits, I'm saying, it might get a bit long, a bit difficult to navigate in the first time. Maybe, maybe I dunno, because now if, if you see the top bar, you see where you're going, you see how long your journey is. But apart from that, I think it's, it's extremely useful.	What I'm not finding straight away is… okay, this is for those who have this mutation. The top one is the mutation and the bottom one is people without the mutation.	I think it's very good to visualise it, because, numbers don't yeah. Risk numbers at the end of the day, you see so many <laugh>, it's difficult to kind of grasp what you mean, but when you see a visual, I think it helps.	Add navigation pane showing where people have been on the website.<br>Make '*MLH1*' and 'general population' bigger and bolder to emphasise.	Must have
p005	I was just struggling a little to remember what was first. For the variants. Maybe if it's bold or I dunno the name of the organ you're looking at. In relation to the image, maybe that would help. See which one.		It's nice. It's difficult to put age and risk together, but I think with different colours and the way they're explained,it makes sense. It's relatively straightforward.	Add name/illustration of womb and ovaries to explain which cancer icon arrays refer to.<br>Include separate dropdown for womb and ovarian.	Must have
p006	[Interviewer: If you scroll down, there's different sections here, you can expand showing the choices.] See, and so that's			Add next button at top as well as bottom so people don't miss it if they forget to scroll down	Should have

	really interesting that you just said that because it's the way I am. I mean I just jumped, I didn't even scroll, I didn't know. So again, sometimes folks will put those buttons both at the bottom or at the top as well so that you forced to see it. Um, I didn't realise that. I mean I could see that there's a story if I click along here but I didn't realise I could do it it by doing this.				
p007	The advice is you have a hysterectomy if you're between 35 and 40 or above. And then I was going down the other and I thought this was a much more telling graph for me than that one up the one at the top where 75 was the dividing line and the advice is 35, 40. I think the one saying below 75, above 75 was a bit... You know, everybody's reading this who, who's then rests further from age 35, 40 I, you should have considering a hysterectomy where they'll be thinking, I've got a 50% chance of cancer from 35, 40.	I've not looked it on the mobile actually. Uh, yeah, so it does work there as well.	I think this tells a better story saying your risks start to appear at 35, 40 and it's something you should consider. [Interviewer: So you liked the one with the coloured people showing by which age?] Yeah. I think I like this representation of it building out. I think that's a much more useful representation of risk.	Make it clearer that up to age 75 means risk over a person's whole lifetime, not 50% risk by age 35/40. Include section 'How does my chance of cancer change with age?' with age-stratified risks. Include comparison to general population risks.	Should have
p008	Does it discuss the psychological and mental impact and how we deal with that aspect and things like that? I think for me, even more than the physical, it was dealing with the psychological, you know, from that moment that, you know, you've got cancer to the confirmation that was, earth-shattering for me. I didn't know whether I was coming or going. I was like a	Because I think much of the damage that we do to ourselves, we do it psychologic		Signpost to psychological support, GP, genetics. Add a disclaimer at the start of the module that this is for people who do not have a diagnosis of cancer, and are considering risk-reducing surgery. If you've been diagnosed with cancer, this session won't be relevant for you as you'll be deciding on the best treatment for you with your care team.	Must have

	zombie. I think it's more about that period for me that I would have liked some kind of, I dunno, support.	ally anyway.			
p008		You want something that doesn't tell you to do a hundred different things before you get to the answer <laugh>	It's easy to manoeuvre. [about link out to NHS page] Oh, that's amazing. It's going out. Oh yeah. That's nice. Yeah, yeah, yeah. So that's the NHS. That's cool.		N/A
p009		It's interesting that it says you are higher risk if you are older. How old does that go to? Because I don't have some tests anymore because I'm past that age.	I think that's really very, very good. Especially someone young. I think that it takes a lot of scary thoughts away. I think it's set out really sort of idiot-proof. It's direct, not complicated, easy to read, easy to find your questions. I think it's really well set out.	Change to 'In the general population, the chance of womb and ovarian cancer is higher over age 50. This is different for people with Lynch syndrome.'	Must have
p010	Um, so for tissue recovery, so like to sort of be pretty much pain free. A bit more	Pain was controlled		Remove the word 'fully' from the option grid and just say 6-8 weeks to recover	Should have

	than 12 weeks, but yeah, it did take six weeks. [Interviewer: so is it even a bit tender now? (after 12 weeks)] Yes. If I lift something a bit heavy. Um, um, it, it can still, you can still just feel it. It's a bit sore.	but I was super shocked at how they were like, you will be really tired. And that lasted a long time. I slept a lot <laugh>. And I guess your body's just recovering and healing, isn't it?		from the operation.<br>Add 'During this time, you may be more tired than usual, and should rest as much as possible and not lift anything heavy, such as bags of shopping.'	
p010	And do they all, all of those genes all affect the same parts? Sorry, just before I carry on, I just had a thought coming to my mind. So can you carry 2 of those gene or 3 of them or all 4?	I went for a hysterecto my and said why I'm having this is cause I've got Lynch syndrome. And then they'll be like, oh, what's that? A lot	Um, oh, that's interesting. Oh, I'm gonna go and look and I need to find out what I, what one I am. [which gene]	Add reassurance and explanation about the variants as a dropdown here. Usually people have a variant in only one gene.	Should have

		of people don't know what that means.			
p010	I started off with skin patches, which were, fine and handy apart from the annoying kind of sticky <laugh> thing that they leave, gluey stuff which is quite hard to rub off. You need Vaseline. It's interesting how she's using it now on her [lower] arm, but on the leaflet on my one it said to use on the upper arm, on the outside up to the shoulders, um, out or on my inner thigh. So, I kind of alternate. Um, so not what’s on here.		This is really good. It's really good having it all in like one place.	Add disadvantage about sticky glue from patches being hard to rub off, can use Vaseline. Change illustrations because the patches and gel show it being applied on wrong part of the body (patches should be below the waist, gel upper arm or inner thigh, on hairless skin).	Should have
p011	On the CaPP3 study, the aspirin tablets were enteric-coated and they're not bad for your stomach lining. In critiquing this section, what type of aspirin should I be taking? I have in my bag this one called dispersible; you know the soluble ones. Is that okay for your stomach? I didn't know, like now I'm off the study, obviously the one, the aspirin that you're taking in the study is obviously okay to be taking every single day for the 5 years or 7 I was on it, but now I'm off it and they're no longer sending actually which aspirin is the best one? I dunno.			Add: 'Some people like to take enteric-coated aspirin, which has a special coating to stop it dissolving in your stomach. There is no evidence about whether this helps avoid side effects or not. It’s also fine to use dissolvable aspirin if you prefer. If your GP agrees that it is right for you to take aspirin, you may want to buy your aspirin as that is usually cheaper than having it on prescription, if you pay for prescriptions.'	Must have
p011	The *H. pylori* thing, you just need to take once or is it recommended to you every few years or no….			Explain *Helicobacter pylori* test usually just done once, through the GP.	Must have

p011	I dunno... the wording, like I worried, concerned. like I'm not, so, I don't worry about it. I'm just, I want to do something to reduce my chances of getting it, but on a day-to-day basis, I don't worry about it. [Interviewer: So, for you it wasn't about feeling less worried, it was about I want to do something that will help me lower my chance of getting bowel cancer?] Yeah.	I'm concerned about possible side effects. That matters a little bit actually. I think. If I was filling this in in the first year, I would say matters a lot.	Knowing there's medical evidence, I like that...	Add another line to ' I want to do something that will make me feel less worried…' to say 'I want to do something that will help lower my chance of getting bowel cancer in the future'. Add on the first page that taking aspirin might not be an option for some people, we recognise that this might not be possible for you. Check at the beginning if aged 70 or over.	Must have
p012	I suppose a question I'd ask you... I'm very old school... is there an option here to speak to a human being? I wonder then, and this may be too problematic in the system, may not permit, but in addition to these tiles giving a dropdown of all the helpful information, whether there's a phone number that someone can call if, if you feel that I, I'm just thinking if your question might not be answered…	A 24-hour hotline might be difficult to manage. I'd quite like to speak to someone if I'm anxious. I'm thinking of older people who feel more comfortabl		Explain this is designed to support you to talk to your GP or genetics service. Resource for chatbot or helpline is something to apply for in future.	Would have

		e speaking about something like this to someone with a friendly… I dunno if that was a good idea <laugh> Just an observatio n.			
p012			Very good,presenting the risks, presenting the side effects. So, they can make an informed decision.		N/A
p012	Does it give an option? I mean, is there, is there an option for someone like me or a version for someone can't take the aspirin every day for something else they might have as an aggravation effect?			A lot of people may not be able to take aspirin after cancer surgery. Signpost to lifestyle section as an alternative to reassure that there is still something they can do.	Should have
p012			They did a profile on my [fundraising]. And when I read it, I found it of great comfort, to see that there were other people like me. And different ages. There are people who have great success stories	Include more patient stories to provide hope and encouragement.	Could have

			and sail through it. There are others that have a tough experience. I think this wants to give that balance, to give patients the optimism. And actually, if caught early, it's one of the most solvable.		
p012	Are people going to think that the answers they're giving are absolutely, truly appropriate to them and that the suggestions that pop up will set them on the right journey or actually should, rather than making a self-diagnosis online, but should they not just go and see the GP? This shouldn't be seen as taking the place of speaking to a professional. That's my only thought. I wonder if a disclaimer [is needed] that suggests this is to try and assist and guide and not a fail-safe method of diagnosis. For some people this maybe be very, extremely helpful to kickstart their understanding. You could say, well, that's, that's common sense, but I don't know, to some people, perhaps you need to be a bit more prescriptive.		[about side effects of taking aspirin for a long time] Hey, that's important. But I think, you know, essentially it's extremely informative. I didn't realise it would go into this much detail actually. It makes sense and it’s easy to navigate.	Make box on first page bigger about it preparing you to see GP	Should have
p013	Um, the genetic service, is that a national service? Is that through a GP? So, some information about that would help…			Explain about pathway to request appointment with local clinical genetics service via GP referral.	Must have
p013	[about animated icon array with coloured blue people] Maybe have it so that you	I have not known that		Move up the text that says 'the older you are the more likely you are to have	Must have

	don't have to scroll down so far for that information. [about 'the older you are, the more likely you are to have side effects] I think that this is a useful thing to have highlighted slightly higher. So, you can see it without scrolling.	bruising more easily is a side effect…		side effects'<br><br>Some pages are quite hard to read on the phone, need redesign.	Should have
p013	I have always had difficulty with gender, but I believe that woman was on top because of female, male done alphabetically. And I dunno whether gender or sex relates to male or female or woman and man, and which way around it is. There is a government site, gov.uk, listing the correct conformity, and some additional information. I've used it before, it was a good source of information.			Stakeholder (BC) provided guidance: Gender identity isn't what will alter risk. Agreed to change to: If you would like to see information that is personalised to you, you can answer the following questions. What sex were you assigned at birth*? Female, Male, Prefer not to say *Unfortunately there is not enough data to show personalised information for intersex, trans and gender diverse communities, but you can seek advice from clinicians.	Must have
p013	Some breadcrumbs would be useful. I just thought about that, but I actually don't know where I am on the website or how to get back to this page. Um, cause there's nothing indicating it over, over on this left hand side here or anywhere up here. Um, so, so breadcrumbs are, um, basically like a, um, a route of where you are for certain things.		This is really interesting information. The way it's been split out with graphics and text is really helpful. The blue people is good. And this last one in particular, the stark contrast [about the lower risk in general population].	Add breadcrumbs at the top to aid navigation, as well as the collapsible navigation page on the left.	Must have
p013	It's important to add that point about don't worry, just because you have any of these things, it doesn't mean that you have bowel cancer. Important to give reassurance. There's an element of why			Move to the top to provide reassurance. Add: 'Common symptoms of bowel cancer are listed below. Don't worry as these symptoms are very common for lots of other reasons too,	Must have

	people are coming to this site? They have Lynch or know someone. They are after more information or are concerned. It's about getting the tone correct.			so most people with these symptoms do not have bowel cancer. However, it's worth going to your GP if you notice one of these things, just to check.'	
p013	So with Lynch syndrome, is the chances of bowel cancer specifically is what is being highlighted, not the other additional risks to other types of cancers that are also associated with?			Add: 'Bowel cancer is one of the most common cancers for men and women with Lynch syndrome'. Lead into the second statement about taking aspirin to reduce that chance.' Set the scene: 'This session is about taking aspirin to reduce the chance of bowel cancer. It only talks about how aspirin reduces the chance of bowel cancer, because there is not yet enough evidence about how it affects the chance of other cancers.'	Should have
p014	The trouble is what it says at the bottom: 'However, it's still worth talking to your GP.' I mean, it's my experience and she's lovely, Dr. [ ], but she hasn't got any time to do anything. It's just, there's the joined-up communication and trying to deal with these things seems to be incredibly challenged these days... it's not a UK phenomenan, it's going on everywhere in the developed world.	Yeah, well see, I haven't got asthma, I haven't got anything. Um, no, I haven't got any of those sorts. No things.	It's a great thing that you're doing. I'm just, I suppose I'm so far ahead of most people coming into the Lynch syndrome environment because I've now been living with it for 11 years. Developed and have all these contacts so forth.	Support for GP education needed. Website could double up as an educational resource for healthcare professionals.	N/A
p014	You know, that's what you should be telling people. It's all very well about aspirin, but ask them to have a bowl of blueberries every day? It's an amazing difference to them. The fibre thing is so important. To keep your bowel system	If you believe in your treatment, whether the	I have become infinitely more positive and infinitely more efficient as a human being since I improved my diet, I would say probably	Consider for lifestyle session of website and signposting to mental health support, coping strategies.	N/A

	flowing quickly and efficiently. That's what you should be, um, concentrating on. And that's what the message should be. That's, that's why this, this thing should be guiding people towards…	treatment's right or not, having a positive mental attitude must be 50% of the benefit. I happen to be very positive so that I'm very healthy. But if you aren't, you've lost the battle. You've lost half the battle.	diet, it's probably the, the be all and end of, uh, of, of tackling almost all cancers, including Lynch.		
p015	It will be quite cool if somewhere on here you could print as many pages as you like. We are all different people like me, you know old lags who used to print everything off, congenital, you know, blockers of printers, <laugh>, now we no longer print anything off unless we absolutely have to. But sometimes there's something to be said for the look and feel and the tactile nature of engaging with paper and circling and highlighting stuff,	It's interesting the word 'session'. How will this session help me? As opposed to section, for example,	It's a good [decision suport] session. I think the reason I like the word session, it kind of suggests that it is some kind of info share or this is, you know... A kind of self-contained piece of information that I can then kind of print off or	Make it clear pages can be printed<br><br>Focus group participants preferred the word 'session' to 'section' or 'module' which felt more like they were being tested.	Should have<br><br>N/A

	and then taking it away to a discussion with my counsellor, my GP or whatever. Which isn't necessarily always easy if, you know, if you're looking at stuff on some kind of, you know.	but session always kind of sounds like I'm entering into some kind of webinar. How will this particular part of the programme help me?	or do whatever I want to do.		
p015	I wonder if there's something in here which says there comes a point in time after which you're advised to stop taking aspirin. Because I think that is the case, isn't it? I think that is 75 or something, maybe. I can't remember. I think it's, yeah, because your chance of cancer could rise as you get older. But hang on, you just said that, you know, you're gonna become higher risk. You get older anyway, if I do take aspirin. What you're saying there is, well, actually, that is that, that your chance is also higher if you are overweight or if you eat too much red meat regardless of whether or not you have Lynch syndrome. right? I, I guess, I don't know.	I have Lynch syndrome. Are you saying the, so this is kind of a generic statement anyway, isn't it? So regardless of, I think it's absolutely valid. Sure. I've done this. I'm	That's why I take my two 75 mgs a day, to do what I can to allay the, to reduce the risks. It makes me feel like I'm doing something, I'm taking ownership in whatever ways I can.	Revise language about age-related cancer risk and modifications: 'Your chance of getting bowel cancer would be lower than if you did not take aspirin. Your chance would still be higher than someone in the general population. The chance of bowel cancer would be affected by other things like if you are overweight or eat too much red meat.'<br>Page 3a: Change to 'In the general population, the chance of bowel cancer is higher over 50. This is different for people with Lynch syndrome. You can look at this graph to see how your chance of bowel cancer changes with age." Insert link to PLSD database.	Must have<br><br>Note: These changes apply to the hysterectomy session as well.

		just talking out loud.			
p015	I think perhaps how much aspirin would you need to take. I think a better word to have in there is actually 75 mg is what's generally known as a baby aspirin. Two baby aspirin is not very much. So kind of quantify what we mean by how much aspirin would I need to take? I don't know what a normal dose of aspirin, it's like a pain killer, right? It's 300mg I think.	Beyond signpost, you are hoping people will take decisions that might be beneficial. If your view is that people should be taking aspirin then I think it doesn't hurt to have something in here saying we're not talking about that much aspirin anyway.		More explanation about aspirin dosage. Add: 'A normal aspirin pill sold over the counter for pain relief is 300mg' for context.	Must have
p015	There comes a point when the genetic counsellor may have a lot on their plate	If my GP asks how		Speaking to the GP will allow for a holistic assessment of overall health	N/A

	and you might not necessarily get the time with them that you might need or crave.	much aspirin are you taking, by the way? Yeah, you're taking 600 mg. Really? What, hang on, what do your genetic counsellors say?		and flag up any issues that might mean aspirin is not right for someone.<br>No change needed as we signpost to GP and genetics services already.	
p015	Gene variant. Um, I'm not sure. Interesting. Is that the case? You can only have one variant.			Explain the 4 Lynch genes and that a family will have a variant in one of these genes, if not sure which one you can ask your genetics service. Added this as a change for the 'chances of cancer' session as we're removing this page from the aspirin session	Must have
p015	So my takeaway from that is well, perhaps confusion, if I take aspirin, my chances of getting bowel cancer are reduced from 55% to 7%. I'm pretty sure that's not what you're trying to tell me. The number of people with Lynch syndrome who got bowel cancer over 10 years who did take aspirin. Well, it's only 7 out of a hundred. [Interviewer: Can you see why the numbers are so different, of what it's actually including in the study in the numbers, this one compared to that other	So these images show the average number of people who get bowel cancer in the UK by the age of 75. I suppose		This page shows 10-year risk with icon arrays out of 100, based on CaPP2 study, but previous page had similar icon arrays for lifetime risk up to age 75, and out of 10. Patients struggled to compare the 2 pages because of different denominators and length of time. Agreed to move the chances of cancer pages into a separate session. Added clarification: 'It is important to understand there are some limitations to what we know about aspirin and	Must have

	picture?] These images show the number of people with Lynch syndrome who got bowel cancer over 10 years with and without taking aspirin. That's fine. But that's the only, that's the only difference that I can see. [Interviewer: So that's really helpful because, um, it's only a 10-year period here.] Oh no, I saw the 10 years, but the other...	what this is saying to me is that if I were to take aspirin, my chances of getting bowel cancer will reduce substantially over the course of the next 10 years.		Lynch syndrome: The chance of bowel cancer is different for each Lynch variant, but this study only shows how aspirin works for people with Lynch syndrome in general. There is not enough information to show how aspirin reduces the chance of bowel cancer for different Lynch variants.' and 'The study only shows the chance of cancer over 10 years, because that is how long people were in the study for. However, the chance of bowel cancer over your whole life up to the age of 75 years will be higher than this. You can see more about your lifetime chance of bowel cancer here.'	
p016	Fine, so I'm sure it [cancer risk] probably increases. I wonder what age group this is looking at as well. Is it just a 10-year period of different ages or…?<br>So it's almost half [50% risk reduction], isn't it? I mean it's still not a massive... I know it's a big lot, but it's… Interesting. I guess that actually only 7 out of the hundred or or even actually only 13 out of the hundred people got bowel cancer who had Lynch syndrome. So... I'm sure I read it was like 70 or 80%... [Interviewer: Can you see anywhere here why the number's lower?] Um, was it a time, was it a time? Over 10 years.			Explain the CaPP2 study of daily aspirin included patients with a range of ages, all adults.<br>Make it clearer that the figures refer to cancer risks over a 10-year period.	Must have
p016	Yeah...I had the chat, I went to the doctor... I got the [aspirin] prescription,		That just shows next steps for you. Yep.		N/A

	I've got it sitting in my cupboard...so the want is there or the desire to do it, but I, you know, cause I'm so early in the stages I suppose I've just had it all removed I think cause I'm being screened so much and you know, I know it's a relatively slow growing type of cancer. If it wasn't, it would come up every 18 months. So then do I need to be doing that now as well? I dunno... Surely, I'm not gonna get it again.		That's good. Yeah, I think that would encourage someone to go to the doctor. Chat about it even though they don't know. Reading all the studies I suppose as well.		
p017	This is interesting because I did the CaPP3 study, and they've said to me to continue until I'm 70.			Explain some people may take aspirin longer, up to age 70	Should have
p017	When you are talking to professionals and when I've spoken to [genetic counsellor], so much of what you talk to others about is about how to live and how to respond medically, but actually to talk to family. When I saw that there, that was the bit that I need the help with, if anything. Does that make sense?	You are obviously doing things to educate GPs and because also, you know, the genetics service, I mean they, they, they're absolutely brilliant, but I can imagine it may just must be	I think it's really good actually, I mean there's a lot of information now, which is great, like 10 years ago there was nothing. But this is amazing. I think this is just, you know, because there's so much information here, which probably took me a very long time to get. And this is great 'cause it's all in one place.	N/A<br><br>Consider for talking to family session.<br><br>Further support for GP education.	Should have

		snowed under. There's lots of other genetic conditions …			
p018		Do you know the thing that jumped out at me and, um, I suppose maybe it wouldn't have done so until I got my, um, diagnosis, but it was the talking to family, the biggest thing that actually it's like a big elephant in the room a lot of the time.	And so...that's what caught my attention...talking to family was the first thing I flipped on when I came onto your page. But no, it was just reassuring to see that it was even the activity other people were struggling with that. Oh, it's something a family's going through. But actually talking to the family then it has, its, you, you are all trying to be overly sensitive to each other's needs at times.		N/A

p018	I take 75mg, do you think it would be worthwhile talking to my doctor with regards to that now and saying that obviously the, the, um, that the research is highlighting that it might be that I need to take 2 of those a day so that I'm taking 150mg?<br>The other thing I was wondering about was whether or not there's like a minimum age to take as aspirin.		Gosh, that's the sort of thing that makes it so much clearer. You hear all these percentages and you hear it but actually visually you see it like that. I'm a really visual person. You can see the impact it's having if you take the aspirin. Look at that.	Another query about aspirin dosage and taking 75mg despite this not being clear in the guidance. Added clarification. Add to page 1 in the list of criteria for not being able to take aspirin, e.g. Aged under 18	Must have
p018	Makes you stop and actually question those points.<br>Your mind goes into overdrive. And there's so much to consider, especially when you have received that diagnosis.	[about side effects] To me it matters a little, as long as I'm aware of what the symptoms are and I'm mindful.	The pointed, targeted questioning helps you to find your way through That element of clarity over whether or not, those questions help you to think it through properly.<br>[printed summary to bring to clinic] That's really helpful. Cause that just helps you to gain that clarity.	Values-based exercise helpful to deal with information overload and encourage deliberative decision-making.	N/A
p018	What if you're quietly panicking inside and you've just been on the website? Actually having a contact that you could ring? I don't know if there's people you could speak to. You know, I'm fortunate that actually when I've needed somebody to talk to that [genetic counsellor] has been there <laugh>.			Consider funding for longer-term implementation and support: contact details for help with website, clinical queries for a Genetic Counsellor.	Would have

p019	Activity might be a bit patronising… Ah, I don't, I dunno what else I'd use. So a, um, something. But activity sounds like my primary school.		I like that you split it out and the hyperlinks, then it clicks straight to that.	Change 'activity' to 'Support to help you think about what's important to you'	Should have
p019	I think I'd like to know a bit more about why aspirin seems to reduce bowel cancer. Even it's just a very brief: studies have shown it does that otherwise it seems to jump straight into, there's not enough evidence. I'm like, okay, why are you, why are you asking me to take it? Don't say not known. Because people might, I dunno, you might get people who are very sceptical be like, oh well if you don't know why am I taking this?	[about picture of bowel] I'd include that somewhere else. I feel like if I'm already looking, I guess aspirin is more specific to bowel cancer, um, you maybe make it a bit more scientific of how we think it does it.	You see, I'd say some of this is some of the info I'd want to know straight up. I think these are the headlines I'd want to know. I think dropdowns are a nice format 'cause it seems nice and tidy. And yeah, I prefer that dropdown format actually to the other page.	Maybe we could change the bullet 'It only looks at bowel cancer, because there is not yet enough evidence about whether aspirin reduces the chance of other cancers related to Lynch syndrome'. to 'Aspirin has been shown to reduce the chance of bowel cancer. There is not yet enough evidence about whether aspirin reduces the chance of other cancers related to Lynch syndrome.'<br>Also add this sentence to the diagram of the bowel, to explain why it's there.	Should have
p019	[about links to other pages being elsewhere on website] I'd keep it actually because people might not read the whole website.	These are statistics and these are charities that can		Add a link to NHS page on bowel cancer here. If you want to read more about treating bowel cancer, click here: https://www.nhs.uk/conditions/bowel-cancer/treatment/<br>Charities in help and support session.	Must have

		help. Just a couple of hyperlinks I think would be really nice.			
p019	Like yes you have Lynch but here are some other things and aspirin may may not control some of these.			Move 'What affects my chance of bowel cancer' dropdown to 'more details' page, rename: 'If I don't take aspirin, what else can I do to lower my chance of bowel cancer?'<br>Show 3 things that can't be changed, and things that can be changed. Add: 'There is not yet enough evidence about how much these changes will lower your chance of bowel cancer in people who have Lynch syndrome. Most of the evidence is in the general population.'<br>Add: 'There are other things you can do that may lower your chance of bowel cancer, like eating less red meat, drinking less alcohol, and keeping a healthy body weight. You can read more here.'	Could have
p019	[anything to add?] Maybe what to do if you have any of these issues, how quickly you should speak to a GP? What kind of things to say that kind of escalates that priority? Is there a link to CaPP2?			Add what to do if you're worried about symptoms: see your GP, link back to symptoms page.<br>Add link to CaPP2/CaPP3 study.<br>Add link: 'This website might help your GP to understand the evidence behind the guidelines about aspirin': https://www.nice.org.uk/guidance/ng151/resources	Must have

p020	Might be a bit better colour contrast perhaps, ‘cause some people might not see much difference between the 2 colours. I mean, I can see… Maybe if they're bolder or something like that, so it's perhaps not so dependent on the colour.	I think it's [age-stratified icon array] quite personalised isn't it? This, what's right for you is not necessarily right for some somebody else	[Interviewer: Is it clear what the (icon array) is trying to show with the numbers?] Um, yeah. Yeah, I think so.	Make colours consistent with hysterectomy session, as people liked that.	Could have
p020		Without the actual CaPP3 results, I can't really say what the right amount is.	Alright, so then you have you, then you have the link to the evidence. Yeah.		N/A
p020	Maybe say a little... more for the aspirin, ‘cause I'd be probably... a bit more worried about sort of stomach impacts of taking a lot of, lots of aspirin.			Include a link to the side effects page when hovering over 'side effects', for people who may not have read this or want to remind themselves what the side effects are	Should have

Table S3. Additional quotes from think-aloud interviews used to construct overarching themes and subthemes to inform optimisation of Lynch Choices to overcome barriers to engagement and promote shared decision-making.

**OVERARCHING THEME:** narrative summary	**Subtheme**	**Exemplar patient quotes from n=20 interview transcripts**
**1. INTERPRETING GENE-SPECIFIC CANCER RISKS AND 'WHAT DOES IT MEAN TO ME?':** People with Lynch were often unaware about current gene-specific clinical guidelines for cancer risk management and which gene was relevant to their family. They found the personalised, age-stratified visual presentations of cancer risks in Lynch Choices helpful to improve understanding and place their risk into context with other life factors such as age.	**1.1 Gene-specific cancer risks are not well understood**	'I didn't realise, depending on which type of Lynch [which gene], you could have just your womb out or they only take the ovaries.' (p001)<br>'I don't even know. I don't even have my gene there, so that would stump me. Like I feel like mine is none of those. Like, I feel like if you were to, if mine was in the list, I'd recognise it, but it's not any of those.' (p004)<br>'Do all of those genes all affect the same parts? Sorry, just before I carry on, I just had a thought coming to my mind. So can you carry 2 of those gene or 3 of them or all 4?' (p010)<br>'Gene variant. Um, I'm not sure. Interesting. Is that the case? You can only have one variant.' (p015)
	**1.2 Age-stratified cancer risks help to put decision-making into context with other competing priorities**	I thought this was a much more telling graph for me than that one up the one at the top where 75 was the dividing line [looking at age-stratified risk graph vs absolute lifetime risks of endometrial and ovarian cancers]... the advice is [to have hysterectomy at age] 35, 40. I think the one saying below 75, above 75 was a bit... You know, everybody's reading this who, who's then further from age 35, 40 [it wouldn't be relevant for older people]... e they'll be thinking, I've got a

		50% chance of cancer from 35, 40.' (p006)<br>'It's interesting that it says you are higher risk if you are older. How old does that go to? Because I don't have some tests anymore because I'm past that age.' (p009)<br>'So these images show the average number of people who get bowel cancer in the UK by the age of 75. I suppose what this is saying to me is that if I were to take aspirin, my chances of getting bowel cancer will reduce substantially over the course of the next 10 years.' (p015)<br>'I guess that actually only 7 out of the hundred or or even actually only 13 out of the hundred people got bowel cancer who had Lynch syndrome. So... I'm sure I read it was like 70 or 80%... [Interviewer: Can you see anywhere here why the number's lower?] Um, was it a time, was it a time? Over 10 years.' (p016)<br>'I think it's [age-stratified icon array] quite personalised isn't it? This, what's right for you is not necessarily right for some somebody else.' (p020)
	**1.3 Visual presentations including pictures and icon arrays were useful and people had preferences, for example about shape, colour and lifetime vs 10-year risks**	I like this. [Prompted: What do you like about it?] It's easy to read, specific to MLH1, comparing to the average population, so you can see how much more is by having Lynch. And it's split between womb and ovarian. That’s important.' (p001)<br>'[Interviewer: so you find it helpful to have it broken down a bit more by age?] I think so. If I didn't have that to refer to then [I would only be able to look at] 4 out of 10 in the lifetime. Some people might live to 50 or 60, might not get to

		80, but for the people who will live that bit longer, have that longevity. It probably is a bit more reassurance to look at it from that point of view.' (p002)<br>'I know it sounds petty, but the colours could be a bit more vibrant. I think more vibrant the colours that stands out more. I like the blue and the lime green, but I think the pink needs to be like a, a shocking pink. So, it's, it stands out. [Interviewers: What about the grey for people not expected to develop cancer?]: Yeah, that's fine. I just think the colours pop out the stronger they are. They pop out.' (p002)<br>'It's difficult to put age and risk together, but I think with different colours and the way they're explained, it makes sense.' (p005)<br>'The way it's been split out with graphics and text is really helpful. The blue people is good. And this last one in particular, the stark contrast [about the lower risk in general population].' (p013)<br>'So my takeaway from that is well, perhaps confusion, if I take aspirin, my chances of getting bowel cancer are reduced from 55% to 7%. I'm pretty sure that's not what you're trying to tell me. The number of people with Lynch syndrome who got bowel cancer over 10 years who did take aspirin. Well, it's only 7 out of a hundred. [Interviewer: Can you see why the numbers are so different, this one (10-year risks) compared to that other (lifetime risks) picture?]' (p015)<br>'Gosh, that's the sort of thing that makes it so much clearer. You hear all these percentages and you hear it but actually visually you see it like

		that. I'm a really visual person. You can see the impact it's having if you take the aspirin. Look at that.' (p018)<br>'Might be a bit better colour contrast perhaps, 'cause some people might not see much difference between the 2 colours. I mean, I can see… Maybe if they're bolder or something like that, so it's perhaps not so dependent on the colour.' (p020)
**2. WORDS MATTER: CAREFUL PHRASING IS IMPORTANT TO FEEL UNDERSTOOD:** People with Lynch had emotive reactions to the words presented in Lynch Choices about risk-reducing options, which prompted consideration of personal values and priorities to encourage deliberative decision-making. It was important for them to feel their rationale for deciding or waiting to decide was reflected in the language used in the decision aid.	**2.1 Choice of words to describe decisions needs to be sensitive and done in partnership with the people who will use the decision aid**	Why is it saying 'Don't have the operation'? So that's little help. It's just the fact that it says 'do not'. It's the only thing I don't like. 'Cause, it's like somebody is telling me 'do not have the operation'. Rather than not have the operation because you were happy. Like if my mom said don't have the operation or something. Like than to not have the operation. [It's] the wording, yeah. But otherwise fine.' (p001)<br>'Or wait to decide later, good to put that in. However, why would you wait in a life-threatening condition? I'm probably reading too much into it, but the options are there and what they mean for you if you don't take it. So I take it if you were to wait to decide later, does that open up to give any further information?' (p002)<br>'Having the operation also makes you feel lots of other things, not necessarily related to cancer. Maybe it should be 'How does this make me feel about my cancer risk 'or something like that? Because it's, it feels like that's a bit of a broad statement perhaps because it's not just about the feelings about, I dunno what you could say instead… Active health, family. All of those

		things.' (p002)<br>'Probably one of the biggest reasons apart from having a family, why I am not choosing to have this yet is ‘cause I really am not looking forward to menopause. And I know that it has horrible side effects from what I've heard. And so that, that would be a huge decision-maker for me. Like I would almost be like, should I just like live with my risk of cancer as opposed to bringing this about early? Like that's a big thing, I think.' (p004)<br>'I dunno.. the wording, like I worried, concerned, like I'm not, so, I don't worry about it. I'm just, I want to do something to reduce my chances of getting it, but on a day-to-day basis, I don't worry about it. [Interviewer: So for you it wasn't about feeling less worried, it was about I want to do something that will help me lower my chance of getting bowel cancer?] Yeah.' (p011)<br>'It's interesting the word 'session'. How will this session help me? As opposed to section, for example, but session always kind of sounds like I'm entering into some kind of webinar. How will this particular part of the programme help me?' (p014)<br>'Activity might be a bit patronising… Ah, I don't, I dunno what else I'd use. So a, um, something. But activity sounds like my primary school.' (p019)<br>'I think I'd like to know a bit more about why aspirin seems to reduce bowel cancer. Even it's just a very brief: studies have shown it does that otherwise it seems to jump straight into, there's

		not enough evidence. I'm like, okay, why are you, why are you asking me to take it? Don't say not known. Because people might, I dunno, you might get people who are very sceptical be like, oh well if you don't know why am I taking this?' (p019)
	**2.2 Symbols and icons are preference-sensitive and benefit from codesign**	I really hate that icon [red circle with line through it], because to me that symbol always means, like, I know you're sick, but I think it's a bit harsh. [Interviewer: Could you say more?] I think it's just like a big red thing in the middle, but you just found out you've got Lynch. You've got to go figure out whether you're going to get yourself into early menopause, or not, in my case. I just feel like it's like you're stopping it, yet you're stopping yourself having surgery and it's like, obviously I would want to do anything. I want to do anything to reduce my chance of cancer, but then also, I want to have a kid, so? I just feel like it’s a bit harsh.' (p001)<br>'So I would say that this text needs to be past the picture. I think the picture’s just too big. I think it's more important to have the text, but yeah, the picture doesn't do anything for me and I think it's really important information about having future children and things like that. It kind of just makes it feel a bit unimportant by having the big picture in the middle.' (p001)<br>'I think that's really powerful. It's kind of scary actually... I knew the risks were higher obviously than the general population, but it's literally just like putting it into this graphic with people [icon

		array showing outlines of people] that really hammers it home.' (p004)
**3. DIGITAL DECISION SUPPORT INTERVENTIONS: THEY CAN HELP BUT MIGHT TRIGGER EMOTIONS:** People with Lynch experienced gaps in information, understanding and support. Lynch Choices could help to fill some of this gap, but also raised questions that were specific to people's personal situation and would be best addressed to the relevant healthcare professional or specialist service. Accessibility to clinical genetics, specialist care, other resources and charities needed improvement.	**3.1 Engaging with the intervention can enhance understanding but may make people feel anxious or confused about their cancer risks and options**	When you're given those sort of statistics, that's really powerful.' (p003)<br>'A lot of people might read this and go, oh my God, I've got cancer. That might not be the case. I mean, I would read that and be like, wow, I have all of those things [symptoms).' (p004)<br>'It's important to add that point about don't worry, just because you have any of these things, it doesn't mean that you have bowel cancer. Important to give reassurance... why people are coming to this site? They have Lynch or know someone. They are after more information or are concerned. It's about getting the tone correct.' (p013)<br>'If you believe in your treatment, whether the treatment's right or not, having a positive mental attitude must be 50% of the benefit. I happen to be very positive so that I'm very healthy. But if you aren't, you've lost the battle. You've lost half the battle.' (p014)<br>'How much aspirin would I need to take? I don't know what a normal dose of aspirin, it's like a pain killer, right? It's 300mg I think.' (p015)<br>'I take 75mg, do you think it would be worthwhile talking to my doctor with regards to that now and saying that the research is highlighting that it might be that I need to take 2 of those a day so that I'm taking 150mg? The other thing I was wondering about was whether or not there's like a minimum age to take as aspirin.' (p018)

		'Makes you stop and actually question those points. Your mind goes into overdrive. And there's so much to consider, especially when you have received that diagnosis.' (p018)<br>'Maybe say a little... more for the aspirin, 'cause I'd be probably... a bit more worried about sort of stomach impacts of taking a lot of, lots of aspirin. ' (p020)
	**3.2 People would benefit from more readily accessible access to specialist information, care and referrals**	The other thing I think that's missing, or not clear, so I hadn't seen a Gynaecologist before and I found that my genetic counsellor, she was like, go see your GP and I kept getting stuck in this loop like. So yeah, I'm not sure if that's the case for every patient. But that for me was, was just a loop.' (p001)<br>'So question about support. It's mentioned speaking to a gynaecologist. I've never spoken to a gynaecologist in my life. I have no idea how to go about being referred apart from ringing my GP and I'm not gonna lie. It's impossible to get an appointment. I've gone to private healthcare because I cannot speak to a GP. If there are other people like me that have moved since their diagnosis, they might be in the same place.' (p004)<br>'I think for me, even more than the physical, it was dealing with the psychological... from that moment that... you've got cancer to the confirmation, that was, earth-shattering for me. I didn't know whether I was coming or going. I was like a zombie... I would have liked some kind of, I dunno, support.' (p008)<br>'I went for a hysterectomy and said why I'm

		having this is cause I've got Lynch syndrome. And then they'll be like, oh, what's that? A lot of people don't know what that means.' (p010)<br>'On the CaPP3 study, the aspirin tablets were enteric-coated and they're not bad for your stomach lining. In critiquing this section, what type of aspirin should I be taking? ...like now I'm off the study... I dunno.' (p011)<br>'The *H. pylori* thing [gastric screening], you just need once or is it recommended to you every few years?' (p011)<br>'The trouble is what it says at the bottom: 'However, it's still worth talking to your GP.' I mean, it's my experience and she's lovely, Dr. [ ], but she hasn't got any time to do anything. It's just, there's the joined-up communication and trying to deal with these things seems to be incredibly challenged these days... it's not a UK phenomenon, it's going on everywhere in the developed world.' (p014)
	**3.3 People highlighted other information and resources like charities and peer support that they found helpful and thought should be signposted**	The [Royal Marsden] Beginner's Guide to Lynch gives [risk figures for endometrial cancer] from 50 to 80 years and the percentage.' (p002)<br>'Is there any connection to Lynch syndrome [referring to Lynch Syndrome UK patient charity] on the page as well? I know it's your booklet, but is there any reference to that as well?' (p002)<br>'It would be really important to link to other external sites, like NHS.' (p004)<br>'These are statistics and there are charities that can help. Just a couple of hyperlinks I think would be really nice.' (p019)

	**3.4 Links to Clinical Genetics Services may be lost over time and need review**	I don't even know who my genetics service are. I haven't spoken to them in 15 years. Now I live in a complete different part of the country, I don't know who my local genetic service would be. I'm probably not like on the books. I've never spoken to them. So, I, I don't even know how I would do that.' (p004)<br>'Um, the genetic service, is that a national service? Is that through a GP? So, some information about that would help…' (p013)<br>'There comes a point when the genetic counsellor may have a lot on their plate and you might not necessarily get the time with them that you might need or crave.' (p015)<br>'You are obviously doing things to educate GPs and because also, you know, the genetics service, I mean they, they, they're absolutely brilliant, but I can imagine it may just must be snowed under. There's lots of other genetic conditions…' (p017)
	**3.5 Lynch Choices as a resource was helpful and valued by people with Lynch**	It's easy to manoeuvre.' (p008)<br>'I think that it takes a lot of scary thoughts away. I think it's set out really sort of idiot-proof. It's direct, not complicated, easy to read, easy to find your questions. I think it's really well set out.' (p009)<br>'This is really good. It's really good having it all in like one place.' (p010)<br>'Knowing there's medical evidence, I like that...' (p011)<br>'Very good, presenting the risks, presenting the side effects. So they can make an informed decision.' (p012)<br>'I found it of great comfort to see that there were

		other people like me... different ages... great success stories and sail through it. There are others that have a tough experience. I think this wants to give that balance, to give patients optimism. And actually, if caught early, it's one of the most solvable.' (p012)<br>'Essentially, it's extremely informative. I didn't realise it would go into this much detail actually. It makes sense and it's easy to navigate.' (p012)<br>'It's a great thing that you're doing... I suppose I'm so far ahead of most people coming into the Lynch syndrome environment because I've now been living with it for 11 years... have all these contacts so forth.' (p014)<br>'[The decision support session] is a kind of self-contained piece of information that I can then kind of print off or or do whatever I want to do.' (p015)<br>'I think it's really good actually, I mean there's a lot of information now, which is great, like 10 years ago there was nothing. But this is amazing. I think this is just, you know, because there's so much information here, which probably took me a very long time to get. And this is great 'cause it's all in one place.' (p017)<br>'It was the talking to family, the biggest thing that actually it's like a big elephant in the room a lot of the time... that's what caught my attention...talking to family was the first thing I flipped on when I came onto your page. But no, it was just reassuring to see that it was even the activity other people were struggling with that.' (p018)

		I suppose a question I'd ask you... I'm very old school... is there an option here to speak to a human being? I wonder then, and this may be too problematic in the system, may not permit, but in addition to these tiles giving a dropdown of all the helpful information, whether there's a phone number that someone can call if, if you feel that I, I'm just thinking if your question might not be answered…' (p011)<br>'A 24-hour hotline might be difficult to manage. I'd quite like to speak to someone if I'm anxious. I'm thinking of older people who feel more comfortable speaking about something like this to someone with a friendly… I dunno if that was a good idea <laugh> Just an observation.' (p011)<br>'This shouldn't be seen as taking the place of speaking to a professional... I wonder if a disclaimer [is needed] that suggests this is to try and assist and guide and not a fail-safe method of diagnosis. For some people this maybe be very, extremely helpful to kickstart their understanding. You could say, well, that's, that's common sense, but I don't know, to some people, perhaps you need to be a bit more prescriptive.' (p012)<br>'That just shows next steps for you. Yep. That's good. Yeah, I think that would encourage someone to go to the doctor. Chat about it even though they don't know. Reading all the studies I suppose as well.' (p016)
	**3.6 Digital decision support resources should ideally include a link to speak to someone for help, for example via a hotline**	'What if you're quietly panicking inside and you've just been on the website? Actually having a contact that you could ring? I don't know if

		there's people you could speak to. You know, I'm fortunate that actually when I've needed somebody to talk to that [genetic counsellor] has been there <laugh>.' (p018)<br>[anything to add?] Maybe what to do if you have any of these issues, how quickly you should speak to a GP. What kind of things to say that kind of escalates that priority?' (p019)
